# Subtyping Burkitt Lymphoma by DNA Methylation

**DOI:** 10.1002/gcc.70042

**Published:** 2025-04-07

**Authors:** Selina Glaser, Rabea Wagener, Helene Kretzmer, Cristina López, Maria Joao Baptista, Susanne Bens, Stephan Bernhart, Kishor Bhatia, Arndt Borkhardt, Shaymaa Elgaafary, Steve Hoffmann, Daniel Hübschmann, Michael Hummel, Wolfram Klapper, Julia Kolarova, Markus Kreuz, Stefano Lazzi, Markus Löffler, Jose Tomas Navarro, Janet Neequaye, Noel Onyango, Timothy Onyuma, German Ott, Bernhard Radlwimmer, Marius Rohde, Andreas Rosenwald, Maciej Rosolowski, Matthias Schlesner, Monika Szczepanowski, Gustavo Tapia, Wilhelm Wößmann, Ralf Küppers, Lorenz Trümper, Lorenzo Leoncini, Peter Lichter, Coral del Val, Ole Ammerpohl, Birgit Burkhardt, Sam M. Mbulaiteye, Reiner Siebert, ICGC MMML‐Seq Consortium; MMML Project

**Affiliations:** ^1^ Institute of Human Genetics Ulm University and Ulm University Medical Center Ulm Germany; ^2^ Institute of Human Genetics Christian‐Albrechts‐University Kiel and University Hospital Schleswig‐Holstein Kiel Germany; ^3^ Department of Genome Regulation Max Planck Institute for Molecular Genetics Berlin Germany; ^4^ Digital Health Cluster, Hasso Plattner Institute for Digital Engineering, Digital Engineering Faculty University of Potsdam Potsdam Germany; ^5^ Hematopathology Section, Pathology Department Hospital Clínic de Barcelona Barcelona Spain; ^6^ Josep Carreras Leukaemia Research Institute Badalona Spain; ^7^ Interdisciplinary Center for Bioinformatics University of Leipzig Leipzig Germany; ^8^ Bioinformatics Group, Department of Computer University of Leipzig Leipzig Germany; ^9^ Division of Cancer Epidemiology and Genetics National Cancer Institute, National Institutes of Health Bethesda Maryland USA; ^10^ Department of Pediatric Oncology, Hematology and Clinical Immunology, Medical Faculty Heinrich‐Heine University Duesseldorf Duesseldorf Germany; ^11^ Faculty of Biosciences, Leibniz Institute on Aging‐Fritz Lipmann Institute (FLI) Friedrich Schiller University Jena Jena Germany; ^12^ Pattern Recognition and Digital Medicine Group (PRDM) Heidelberg Institute for Stem Cell Technology and Experimental Medicine (HI‐STEM) Heidelberg Germany; ^13^ Charité Center for Biomedicine (CC4) Charité—University Medicine Berlin Berlin Germany; ^14^ Hematopathology Section, Institute of Pathology Christian‐Albrechts‐University Kiel Germany; ^15^ Institute for Medical Informatics Statistics and Epidemiology University of Leipzig Leipzig Germany; ^16^ Department of Medical Biotechnology University of Siena Siena Italy; ^17^ Department of Hematology, Institut Català d'Oncologia, Germans Trias i Pujol University Hospital Universitat Autònoma de Barcelona Badalona Spain; ^18^ Department of Child Health University of Ghana Medical School Accra Ghana; ^19^ Department of Medical Microbiology and Immunology University of Nairobi Nairobi Kenya; ^20^ Kenyatta National Hospital Nairobi Kenya; ^21^ Department of Clinical Pathology Robert‐Bosch Krankenhaus, and Dr. Margarete Fischer‐Bosch Institute of Clinical Pharmacology Stuttgart Germany; ^22^ Division of Molecular Genetics German Cancer Research Center (DKFZ) Heidelberg Germany; ^23^ Department of Pediatric Hematology and Oncology Justus‐Liebig‐University Giessen Giessen Germany; ^24^ Institute of Pathology University of Würzburg Würzburg Germany; ^25^ Biomedical Informatics, Data Mining and Data Analytics, Faculty of Applied Computer Science and Medical Faculty University of Augsburg Augsburg Germany; ^26^ Clinic of Internal Medicine II, Hematology Laboratory Section University Hospital Schleswig‐Holstein Campus Kiel Kiel Germany; ^27^ Department of Pathology, Germans Trias i Pujol University Hospital Universitat Autònoma de Barcelona Badalona Spain; ^28^ NHL‐BFM Study Centre and Pediatric Hematology and Oncology University Medical Center Hamburg‐Eppendorf Hamburg Germany; ^29^ Institute of Cell Biology (Cancer Research) University of Duisburg‐Essen, Medical School Essen Germany; ^30^ German Cancer Consortium (DKTK) Essen Germany; ^31^ Department of Hematology and Oncology Georg‐August‐University of Göttingen Göttingen Germany; ^32^ Department of Computer Science and Artificial Intelligence, Andalusian Research Institute in Data Science and Computational Intelligence (DaSCI) University of Granada Granada Spain; ^33^ Instituto de Investigación Biosanitaria Ibs.GRANADA Complejo Hospitales Universitarios de Granada/Universidad de Granada Granada Spain; ^34^ German Center for Child and Adolescent Health (DZKJ) Ulm Germany; ^35^ Airway Research Center North Member of the German Center for Lung Research (DZL) Grosshansdorf Germany; ^36^ Pediatric Hematology and Oncology University Hospital Muenster Muenster Germany

**Keywords:** Africa, Burkitt lymphoma, DNA methylation, Epstein–Barr virus, immunodeficiency

## Abstract

Burkitt lymphoma (BL) is an aggressive germinal center B‐cell‐derived malignancy. Historically, sporadic, endemic, and immunodeficiency‐associated variants were distinguished, which differ in the frequency of Epstein–Barr virus (EBV) positivity. Aiming to identify subgroups based on DNA methylation patterns, we here profiled 96 BL cases, 17 BL cell lines, and six EBV‐transformed lymphoblastoid cell lines using Illumina BeadChip arrays. DNA methylation analyses clustered the cases into four subgroups: two containing mostly EBV‐positive cases (BL‐mC1, BL‐mC2) and two containing mostly EBV‐negative cases (BL‐mC3, BL‐mC4). The subgroups BL‐mC1/2, enriched for EBV‐positive cases, showed increased DNA methylation, epigenetic age, and, in part, proliferation history compared to BL‐mC3/4. CpGs hypermethylated in EBV‐positive BLs were enriched for polycomb repressive complex 2 marks, while the CpGs hypomethylated in EBV‐negative BLs were linked to, for example, B‐cell receptor signaling. EBV‐associated hypermethylation affected regulatory regions of genes frequently mutated in BL (e.g., *CCND3*, *TP53*) and impacted superenhancers. This finding suggests that hypermethylation may compensate for the lower mutational burden of pathogenic drivers in EBV‐positive BLs. Though minor, significant differences were also observed between EBV‐positive endemic and sporadic cases (e.g., at the *SOX11* and *RUNX1* loci). Our findings suggest that EBV status, rather than epidemiological variants, drives the DNA methylation‐based subgrouping of BL.

## 1 Introduction

Burkitt lymphoma (BL) is an aggressive B‐cell lymphoma characterized by a germinal center B‐cell (gcBC) phenotype and a high proliferation rate (Ki‐67 > 95%) [1]. The genetic hallmark of BL is the translocation of the *MYC* oncogene into the vicinity of an immunoglobulin locus enhancer, which leads to its constitutive expression. BL exhibits a stable karyotype with few chromosomal imbalances [2, 3]. The mutational landscape is characterized by alterations in genes involved in B‐cell receptor (e.g., *ID3*, *TCF3*) and sphingosine‐1‐phosphate (e.g., *RHOA*, *GNA13*, *PDGFRB*, *S1PR1*) signaling, SWI–SNF chromatin remodeling (e.g., *SMARCA4*, *ARID1A*), and cell survival and proliferation (e.g., *CCND3*, *TP53*, *USP7*, *RFX7*) [4, 5, 6].

Historically, BL has been grouped into three epidemiological variants: endemic (eBL), sporadic (sBL), and immunodeficiency‐associated BL (iBL) [7]. While these variants share hallmark features like phenotype and presence of *IG*::*MYC* translocation, they differ in geographical distribution, Epstein–Barr virus (EBV) association, age, and anatomical presentation. With eBL being predominantly EBV‐positive, less than 30% of sporadic cases show EBV positivity [7]. Recent data suggest that differences in the molecular architecture of the *IG*::*MYC* fusion and the mutational landscape of BL are more correlated to the EBV status of the tumor than the geographic origin or age at diagnosis of the patient [5, 8, 9, 10, 11]. A study based on integrating data from BL and diffuse large B‐cell lymphoma suggested the existence of three genetic subgroups within BLs: DGG (*DDX3X*, *GNA13*, *GNAI2*), IC (*ID3*, *CCND3*), and Q53 (quiet *TP53*)‐BL, indicating hitherto uncharacterized molecular diversity of BL [6].

Genome‐wide DNA methylation (DNAme) studies in BL are yet limited. Kretzmer et al. analyzed the DNA methylome of sBL and showed that DNAme and somatic mutations cooperatively deregulate key B‐cell pathways [12]. Studies on eBL generally report high DNAme levels at CpG islands and promoters, potentially silencing tumor suppressor genes [13]. Nevertheless, those studies are mostly limited by targeted gene analyses, a low number of samples, or reliance on BL‐derived cell lines, which may not recapitulate findings in primary biopsies. Therefore, we aimed to identify epigenetically distinct subgroups of BL via joint analysis of genome‐wide DNAme data from primary biopsies obtained from all three BL epidemiological variants.

## 2 Materials and Methods

### 2.1 Patient Material

The sBL samples (*n* = 80) were collected within the studies of the “Molecular Mechanisms in Malignant Lymphoma (MMML)” network as the MMML‐, MMML‐MYC‐SYS, and ICGC MMML‐Seq projects, which have been approved by the ethics committees of the coordinating and required recruiting centers (e.g., D474/14, D447/10, 403/05, A150/10 Ethics Committee Medical Faculty of the University of Kiel; 349/11 Ulm University). Cases submitted as sBL to the MMML‐projects were reviewed by an expert hematopathology panel, selected for high‐tumor cell content (> 60%), and presence of an *IG*::*MYC* translocation by fluorescence in situ hybridization or by whole genome sequencing, and absence of break targeting *BCL2* and *BCL6*. Available clinical data on the sBL cases do not allow to rule out an underlying immunodeficiency in all cases. Each case turned out post hoc to be human immunodeficiency virus (HIV) positive and to have an inborn error of immunity [4].

The eBLs (*n* = 29) entering the study were recruited in the framework of the National Cancer Institute's Ghana Burkitt Tumor Project between 1975 and 1992 and stored long‐term under liquid nitrogen vapor at the Frederick National Cancer Laboratory in Frederick, MD [14, 15]. The samples were obtained from abdominal masses (ovaries, kidney, or spleen) before treatment and were diagnosed based on local cytology or histology. Most samples were collected before the onset of the HIV pandemic, so most are presumed HIV negative. These samples were previously investigated for chromosomal translocations and other abnormalities [16]. The NIH Office of Human Subject Research Protection gave ethical approval to use the Ghana samples as nonhuman subject research because they were not linked to any personal identifiers (Exempt #: 4055). BL cases in Ghana are considered endemic and etiologically related to *Plasmodium falciparum* infection based on geographical co‐clustering. Children are exposed to *P. falciparum* infection from birth and typically suffer hundreds of infections per year [17], thus, all children are assumed to have been exposed before diagnosis. The presence of *P. falciparum* infection at the time of diagnosis was not consistently recorded. As controls, we included splenic samples from splenomegalies (*n* = 5) due to chronic malaria infection in African individuals.

A total of seven iBLs were contributed by the Hospital Germans Trias I Pujol/Josep Carreras Leukemia Research Institute (Badalona, Spain) and were all derived from HIV‐positive individuals. They were classified as iBLs based on the criteria outlined by the WHO classification 2016 [7].

### 2.2 Normal B‐ and T‐Cell Populations

Publicly available data from various cell populations covering B‐ and T‐lineage differentiation, as well as macrophages and monocytes, were mined [12, 18, 19, 20, 21, 22, 23, 24, 25, 26].

### 2.3 Cell Lines

DNAme profiles of 23 human cell lines, consisting of six lymphoblastoid cell lines (LCLs) and 17 BL‐derived cell lines (Table S1) were generated as part of this study. The identity of the cell lines was verified using STR profiling.

### 2.4 Determination of EBV Status

The EBV status of the primary tumor samples was analyzed by different methods, including immunohistochemistry for EBNA1, in situ hybridization for EBER, PCR and/or Sanger sequencing for EBV genomic sequences, and/or bioinformatic detection of viral genes from RNA sequencing and/or whole genome sequencing data.

### 2.5 DNA Methylation Analyses

DNA was extracted from fresh/frozen samples in 103 cases and from the used cell lines or formalin‐fixed paraffin‐embedded (FFPE) material in 13 cases. DNAme profiling was performed using Infinium HumanMethylation450 and MethylationEPIC BeadChips (Illumina Inc., San Diego, CA, USA), and the resulting data were processed using the minfi package (v1.44.0) with Illumina‐like normalization in R (against intrinsic controls, without background correction) [27]. A subset of the data has been included in previous studies [3, 4, 12, 15]. Beta values, which represent the percentage of methylation, were calculated. We excluded rs loci, gonosomal loci, and loci with a detection *p* > 0.01. We integrated datasets from the Infinium HumanMethylation450 and MethylationEPIC BeadChips, resulting in a unified dataset containing 441 870 CpG sites. In addition, loci associated with geographic differences (5648 CpGs; for details, see Supporting Information) were excluded. Further, due to a comparatively poorer quality in iBLs compared to the other samples, we excluded CpGs with a detection *p* > 0.01 in iBLs (127 144 CpGs). Finally, 309 078 CpGs were retained for downstream analyses. Additionally, a DNAme‐based purity classifier taking into account the tumor cell content and poised promoter methylation was developed and applied to assess sample purity (Figure S1) [28]. Based on this classifier, 10 sBL, nine eBL, and one iBL samples were excluded due to low tumor cell content. For details, see Supporting Information.

### 2.6 Sequencing Data

RNA‐sequencing data from five gcBCs and 21 solid sBLs were obtained from the ICGC MMML‐Seq project and processed as described in López et al. [4].

### 2.7 Statistical Analyses

All statistical analyses were performed in R (version 4.3.0), unless otherwise specified. The proliferation history was analyzed using the epiCMIT tool in R [29]. The epigenetic age was determined through the Horvath clock utilizing the methylclock package (version 1.6.0) [30]. To derive various purity scores from DNAme data, we employed the following R packages: InfiniumPurify (version 2.0) [31], RFPurify (version 0.1.2) [32], Flow.Sorted.Blood.450k (version 1.38.0) [33], and FlowSorted.BloodExtended.EPIC (version 1.1.2). Consensus partitioning was conducted using the cola package (version 2.6.0) [34]. Differentially methylated CpGs were identified with the limma package (version 3.58.1) [35]. For visualization purposes, we utilized the ComplexHeatmap package (version 2.16.0) [36]. Enrichment analysis was performed via the EnrichR web tool, using genes from the array as the background list.

The Wilcoxon rank sum test was used for pairwise comparisons between independent groups described by continuous variables. For categorical variables, Fisher's exact test was used to calculate odds ratios (OR) and *p* values. As a background set, either the 309 078 CpGs or all samples were used. The Bonferroni method was used to adjust *p* values for multiple tests. Differences with an adjusted *p* < 0.05 (if not otherwise specified) were considered to be statistically significant.

## 3 Results

### 3.1 Study Cohort

Aiming to identify subgroups within BL through DNAme patterns, data from 96 BL cases entered the analysis. Cases were selected for high‐tumor cell content and predominantly studied on cryopreserved materials using Illumina BeadChip arrays. The DNAme‐based tumor cell purity scores were similar across the three BL epidemiological variants despite slightly lower B‐cell composition in iBL cases (Figure S2). The median age at diagnosis was 9 years (range: 2–57) in the 70 sBL cases, 8 years (range: 3–13) in 20 eBL cases, and 45 years (range: 39–57) in six iBL cases. EBV positivity in tested cases was 95% (19/20) in eBL, 50% (3/6) in iBL, and 15% (6/39) in sBL (Table S2).

### 3.2 DNAme‐Based Clustering Is Driven by EBV Status

**FIGURE 1:**
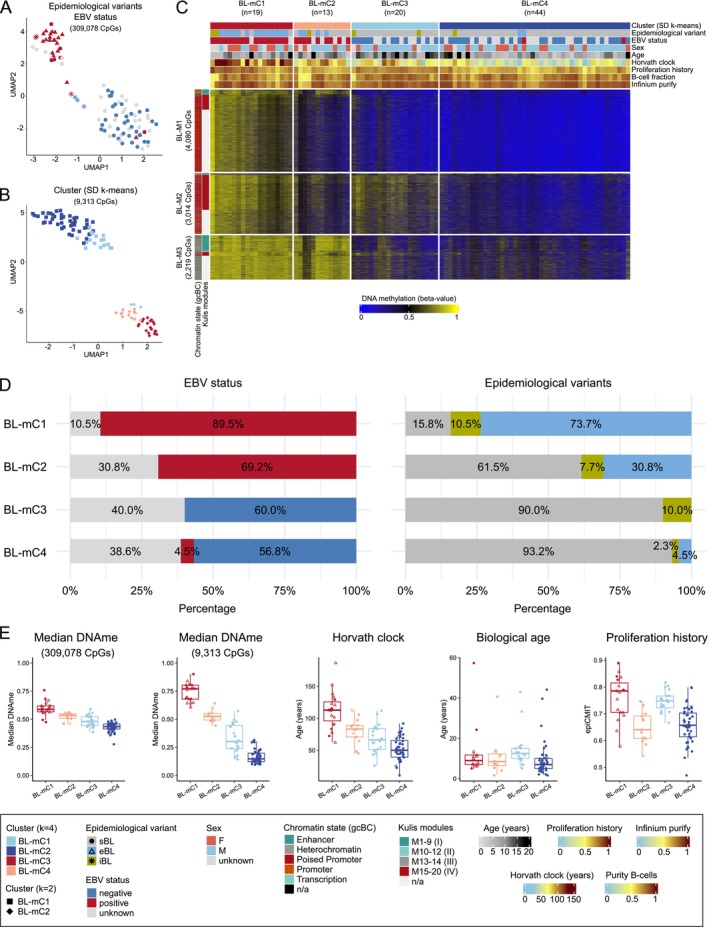
DNA methylation‐based subgrouping of Burkitt lymphoma (BL) cases. (A) UMAP visualization (25 neighbors) of 96 BL cases based on the 309 078 CpGs representing the global DNA methylation landscape. (B) UMAP visualization (15 neighbors) of 9313 CpGs (standard deviation (SD) > 0.25) colored and shaped according to the number of clusters (*k* = 2, *k* = 4) determined by the combination of CpGs filtering using SD and *k*‐means clustering. (C) Heatmap depicting DNA methylation levels of the 9313 CpGs across the 96 BL samples. Columns represent individual samples grouped into four optimal clusters as determined by SD and *k*‐means clustering. Rows represent CpGs, further categorized into three modules (BL‐M1‐3) using *k*‐means clustering. Sample features are annotated at the top of the heatmap, including the four clusters (SD, *k*‐means), epidemiological variants, EBV status, age at diagnosis, epigenetic age based on Horvath clock, proliferation history determined with epiCMIT, B‐cell fraction calculated from DNA methylation data, purity score received from the InfiniumPurify package. CpGs are annotated using chromatin states defined in germinal center B cells (gcBCs) and the Kulis modules, which are grouped according to the patterns (I–IV) in the paper [17] (for details see Supporting Information). (D) Bar plot showing the distribution of epidemiological variants (sporadic, endemic, and immunodeficiency‐associated BL) and EBV status across the four identified clusters. (E) Box plots illustrating key biological and epigenetic features of the clusters, including biological age (age at diagnosis), epigenetic age calculated using the Horvath clock, proliferation history determined using the epiCMIT package, and median DNA methylation levels based on 309 078 CpGs and 9313 CpGs. n/a: not applicable. Statistical comparisons are summarized in Tables S10 and S11.

After excluding CpGs with low quality or associated with geographic differences, unsupervised analysis of the remaining 309 078 CpGs revealed two major clusters that predominantly differed by EBV status (Figure 1A). Next, we employed consensus partitioning methods with different CpG selection strategies [34]. Among the 20 tested combinations, the optimal numbers of clusters identified were two or four (Figure S3, Table S3). Limiting the analysis to 9313 CpGs with a standard deviation (SD) > 0.25 (across all BLs), visualization in a UMAP plot (Figure 1B) revealed two groups that fully agree with the two‐cluster solution from consensus partitioning using SD filtering and *k*‐means clustering (Figure S4). The four DNAme clusters determined by the cola package (SD *k*‐means; BL‐mC1‐4) comprise two clusters containing, with regard to EBV‐tested cases, predominantly cases known to be EBV‐positive (BL‐mC1: 17/17 [100.0%], BL‐mC2: 9/9 [100.0%]) and two clusters mainly consisting of cases known to be EBV‐negative (BL‐mC3: 12/12 [100.0%]; BL‐mC4: 25/27 [92.6%]) (Figure 1C,D, Table S4). Interestingly, BL‐mC1 is primarily composed of EBV‐positive eBL cases (OR: 36.2, *p* < 0.001), while BL‐mC2 contains the majority of EBV‐positive sBL cases (OR: 19.6, *p* = 0.0024).

EBV‐positive BL‐associated clusters BL‐mC1 and ‐mC2 showed higher median DNAme levels for the total 309 078 CpGs than EBV‐negative BL‐associated clusters (BL‐mC1‐2: 0.56 [range: 0.46–0.76] vs. BL‐mC3‐4: 0.44 [range: 0.28–0.59], adj. *p* < 0.001) (Figure 1E). BL‐mC1 and ‐mC2 showed a higher epigenetic age (Horvath clock). For BL‐mC1‐2, we determined a median of 99 (range: 45–186) years vs. BL‐mC3‐4 with 54 (range: 11–112) years (adj. *p* < 0.001). Further, the two EBV‐negative BL‐associated subgroups differed in age at diagnosis (BL‐mC3: 12.5 [range: 5–43] years vs. BL‐mC4: 7 [range: 2–44] years, adj. *p* = 0.007) and also in the proliferation history (epiCMIT) (BL‐mC3: 0.75 vs. BL‐mC4: 0.66, adj. *p* < 0.001). Differences in proliferation history were also observed for the two EBV‐positive BL‐associated subgroups (BL‐mC1: 0.79 vs. BL‐mC2: 0.64, adj. *p* = 0.002) (Figure 1E) [29]. We conclude that DNAme profiles identify two clusters of BL mainly associated with EBV status and that each of these clusters contains two subclusters with cases showing different biological properties.

### 3.3 Properties of CpG Modules Underlying DNAme‐Based Clustering of Burkitt Lymphomas

To uncover potential biological properties underlying the DNAme driving the identified subgroups described above, *k*‐means clustering of the 9313 selected CpGs (Table S5) was performed and revealed three CpG modules: BL‐M1 (4080 CpGs), BL‐M2 (3014 CpGs), and BL‐M3 (2219 CpGs) (Figure 1C). CpGs within BL‐M1 and BL‐M2 are predominantly located within CpG islands (BL‐M1: OR = 3.2, adj. *p* < 0.001; BL‐M2: OR = 2.2, adj. *p* < 0.001) that are defined as poised promoter regions within gcBCs (BL‐M1: OR = 3.2, adj. *p* < 0.001; BL‐M2: OR = 2.5, adj. *p* < 0.001) (Figure S5A). Furthermore, genes associated with these CpGs show significant enrichment for binding sites of SUZ12 (BL‐M1: OR = 6.5; BL‐M2: OR = 10.1; adj. *p* < 0.001) and EZH2 (BL‐M1: OR = 8.3; BL‐M2: OR = 9.6; adj. *p* < 0.001), two key components of the polycomb repressive complex 2 (PRC2) (Figure S5B, Table S6). Genes associated with CpGs in BL‐M3 are enriched for binding sites of e.g., ZBTB7A (OR = 2.3; adj. *p* < 0.001) and GATA1 (OR = 2.8; adj. *p* < 0.001) as well as pathways, for example, related to FOXO (OR = 3.0, *p* = 0.004) and B‐ and T‐cell receptor signaling (OR = 3.7, *p* = 0.004; OR = 3.2, *p* = 0.004) (Figure S5B).

### 3.4 Analysis of the CpG Modules Underlying DNAme‐Based Clustering of Burkitt Lymphomas in the Normal B‐Cell Differentiation

To contextualize the DNAme patterns observed in the BL samples, we analyzed the DNAme levels of the 9313 CpGs in various benign (pre‐)B‐cell subpopulations (Figure S6). CpGs within modules BL‐M1 and BL‐M2 exhibited low DNAme levels in benign (pre‐)B‐cell subpopulations. At the same time, those in BL‐M3 were predominantly highly methylated in benign (pre‐)B‐cell subpopulations. We infer that the CpG module BL‐M3 is characterized by a loss of DNAme in the observed two EBV‐negative BL‐associated clusters BL‐mC3‐4. A subset of CpGs in all three modules displayed B‐cell differentiation‐dependent DNAme changes in benign B‐cell populations. This agrees with the fact that the modules were enriched for CpGs identified by Kulis et al. as dynamically changing during B‐cell differentiation (3848/9313 CpGs, OR = 3.0, *p* < 0.001) (Figure S6) [19].

### 3.5 DNA Methylation Profiling of Burkitt Lymphoma‐Derived Cell Lines and Lymphoblastoid Cell Lines

Next, we interrogated DNAme data of 17 BL‐derived cell lines and six EBV‐transformed LCLs. Independent of their EBV status, BL‐derived cell lines showed high DNAme levels across all three CpG modules (BL‐M1‐3) (Figure S7A). This finding was accompanied by a high epigenetic age (median [range]: 158 years [106–194]) and proliferation history (median [range]: 0.92 [0.85–0.93]) of the BL‐derived cell lines, likely due to the high number of cell cycles they have experienced in cell culture (Figure S7B). Globally, the BL‐derived cell lines predominantly clustered according to the epidemiological variants they derive from (Figure S7C). However, when reduced to the 9313 CpGs, the BL‐derived cell lines formed a separate cluster, probably due to high median DNAme levels (0.61 [range: 0.46–0.79]) (Figure S7D). In contrast, LCLs exhibited lower DNAme levels for BL‐M1 and BL‐M2, forming a separate cluster within a UMAP.

### 3.6 Differential DNAme Analysis Reveals a Hypermethylated Epiphenotype in EBV‐Associated Burkitt Lymphoma

**FIGURE 2:**
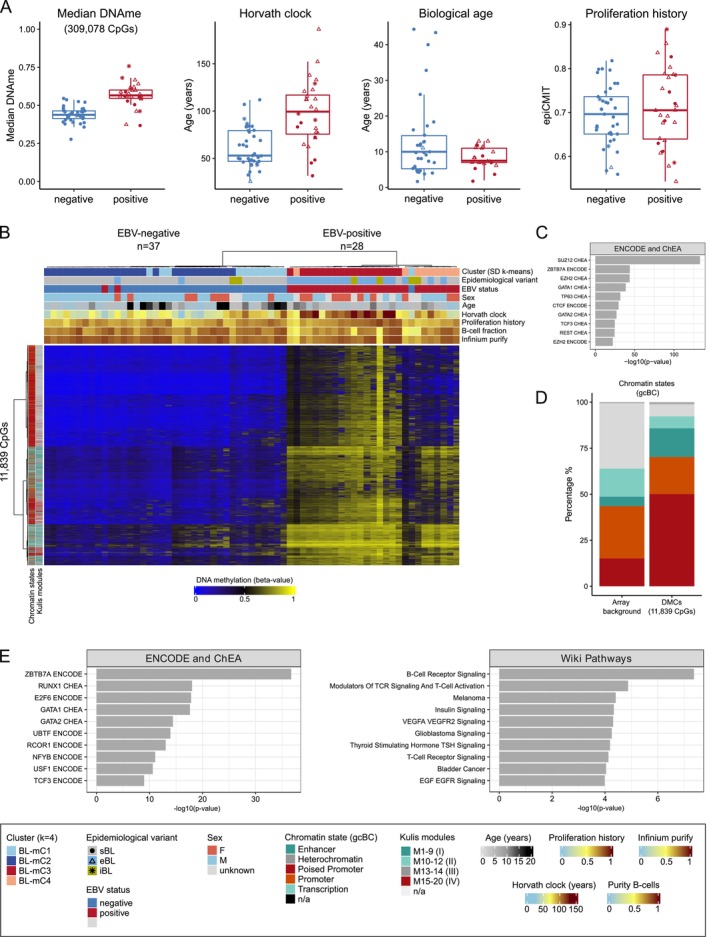
Comparative DNA methylation profiling of EBV‐negative and EBV‐positive Burkitt lymphoma (BL). (A) Box plots comparing EBV‐negative and EBV‐positive BL for biological age (age at diagnosis), epigenetic age calculated using the Horvath clock, proliferation history (epiCMIT), and median DNA methylation levels across 309 078 CpGs. (B) Heatmap depicting DNA methylation levels of 11 839 CpGs found significantly differentially methylated between EBV‐negative and EBV‐positive BL cases (adjusted *p* < 0.01, |Δ*β*| > 0.3, corrected for fixation technique and array). Sample features are annotated at the top of the heatmap, including the four clusters (SD, *k*‐means), epidemiological variants, EBV status, age at diagnosis, epigenetic age based on Horvath clock, proliferation history determined with epiCMIT, B‐cell fraction calculated from DNA methylation data, purity score received from the InfiniumPurify package. Columns represent samples, rows depict CpGs. CpGs are annotated using chromatin states defined in germinal center B cells (gcBCs) and the Kulis modules, which are grouped according to the patterns (I–IV) in the paper [17]. (C) Transcription factor enrichment analysis (based on ENCDOE and ChEA) on the genes associated with the 11 839 DMCs. (D) Bar plot displaying the distribution of the 11 839 DMCs within chromatin states defined in gcBCs. (E) Enrichment analysis of genes using a subset (4416 CpGs) of the 11 839 CpGs (non‐PP2 signature), not associated with binding sites of SUZ12/EZH2 and not located within poised‐promoter regions. Enrichment analysis was performed for ENCODE and ChEA transcription factors, as well as Wiki pathways. The *y*‐axis displays the top 10 most significant gene ontology terms. n/a: not applicable; PP2: poised promoter and polycomb repressive complex 2.

To further explore correlations between EBV status, DNAme levels, and associated epigenetic predictors, we focused our comparisons on confirmed EBV‐positive (*n* = 28) and EBV‐negative (*n* = 37) BLs. Overall, EBV‐positive cases exhibited higher global DNAme levels (median [range]: 0.57 [0.37–0.76] in positive cases vs. 0.44 [0.28–0.55] in negative cases, *p* < 0.001) and an increased epigenetic age (median [range]: 99 [32–186] years in positive cases vs. 53 [26–112] years in negative cases, *p* < 0.001) (Figure 2A). The proliferation history exhibited substantial variability among samples in both groups, in line with the concept that it represents a main distinguishing factor between the two subclusters of the EBV‐positive and negative cases, respectively (Figures 1E and 2A).

Differential DNAme analysis of the 309 078 CpGs revealed 11 839 (Table S7) significantly differentially methylated CpGs (DMCs) between EBV‐positive and EBV‐negative BLs (adj. *p* < 0.01, |Δ*β*| > 0.3), with all but seven of these CpGs being hypermethylated in the EBV‐positive cases (Figure 2B). Analysis of 4829 genes linked to the DMCs showed again a notable abundance of binding sites for SUZ12 and EZH2 (Figure 2C). This finding aligns with the observation that 50.1% of the DMCs are located within poised promoter regions in gcBCs (Figure 2D), potentially reflecting epigenetic footprints of cell divisions occurring within the GC.

To look further into the potential GC‐passage‐driven effects and to elucidate pathways linked more directly to EBV infection, we differentiated the 11 839 DMCs into 7423 linked to 2046 genes associated with SUZ12 and EZH2 binding sites and/or within poised promoter regions (called poised‐promoter/PRC2 = PP2 signature) and those 4416 DMCs not containing these features. These latter 4416 DMCs associated with 2783 genes were mainly hypomethylated in EBV‐negative BLs compared to nonmalignant B cells (non‐PP2 signature) (Figure S8). Functional annotation of the genes in this non‐PP2 signature revealed enrichment of binding sites for ZBTB7A (OR = 2.6; *p* < 0.001) and RUNX1 (OR = 2.4; *p* < 0.001) and genes associated with B‐cell receptor signaling (OR = 4.4; *p* < 0.001) (Figure 2E). An analogous but independent comparison using only the dataset of the BL‐derived cell lines yielded 738 DMCs between EBV‐positive and ‐negative lines showing similar enrichments, for example, for ZBTB7A (OR = 3.4; *p* < 0.001) and RUNX1 (OR = 2.4; *p* = 0.004) target genes and B‐cell receptor signaling pathways (OR = 5.8; *p* = 0.006).

The separate supervised comparisons of the primary BL samples and BL‐derived cell lines regarding the EBV status showed an overlap of 481 CpGs representing 65.2% of the DMCs identified in the cell lines (Figure S9, Table S8). This overlapping set of DMCs included multiple hits for genes involved in lymphomagenesis, like *CD79B* or *TERT*, including several genes linked to superenhancers recently identified in gcBC lymphomas [37].

### 3.7 Differential DNA Methylation at Superenhancers Between EBV‐Positive and ‐Negative Burkitt Lymphoma

The latter finding prompted us to more systematically explore the DNAme levels of 3755 superenhancers (SEs) recently described by Bal et al. in gcBC lymphomas [37]. These SEs are associated with 34 126 CpGs (average coverage per SE: 15 CpGs) in our dataset. Differential DNAme analysis of these 34 126 SE‐associated CpGs between primary EBV‐positive (*n* = 28) and EBV‐negative (*n* = 37) BLs revealed 2407 CpGs (adj. *p* < 0.01, |Δ*β*| > 0.3) involving 1648 SEs (average coverage per SE: 2.4 CpGs) with 22 SEs affected by at least 10 CpGs. While the 22 SEs are highly methylated in EBV‐positive BLs, some SEs exhibit a loss of DNAme in EBV‐negative BLs compared to gcBCs, potentially associated with an activation of the SE (Figure S10).

### 3.8 Differential DNA Methylation According to Geographic Origin Within EBV‐Positive Burkitt Lymphoma and Potential Influence of Malaria Infection

The geographic origin of the tumor is the simplest way to define sBL versus eBL. Based on population genetics differences, we had a priori excluded 5648 CpGs from the analysis to exclude confounding of our results by comparison of samples from Europe and Africa. Nevertheless, we observed differences in the frequency of eBL and sBL in BL‐mC1 versus ‐mC2, enriched for EBV‐positive cases. This finding prompted us to conduct a differential DNAme analysis comparing EBV‐positive sBLs (*n* = 5) and eBLs (*n* = 17). We identified 520 DMCs (adj. *p* < 0.01, |Δ*β*| > 0.2) of those, 497 CpGs show a DNA hypermethylation in eBLs (Figure S11, Table S9). Notably, these included CpGs in regulatory regions of genes like *SOX11* and *RUNX1* [38, 39]. Overall, the CpGs affected by differential DNAme were again enriched for SUZ12 binding sites (OR = 5.4; *p* < 0.001).

The geographic origin strongly correlates with the likelihood of prior exposure to *P. falciparum* infection. It can be reasonably assumed that all individuals with eBL had a history of serious *P. falciparum* infection malaria disease [17]. Therefore, in order to analyze whether the differential DNAme patterns observed in the EBV and geographic groups do not merely represent malaria history, we profiled FFPE samples of malaria‐driven splenomegaly from the eBL region. Comparative DNAme profiling for the 9313 CpGs of the modules, the 11 839 DMCs between EBV‐positive and ‐negative BL, and the 520 DMCs between EBV‐positive sBL and eBL revealed DNAme levels of the malaria‐splenomegaly samples similar to those observed in benign B cells and clearly different from EBV‐positive BL, regardless of origin (Figure S12).

### 3.9 Frequent DNA Hypermethylation of Recurrently Mutated Genes in EBV‐Positive Burkitt Lymphoma

**FIGURE 3:**
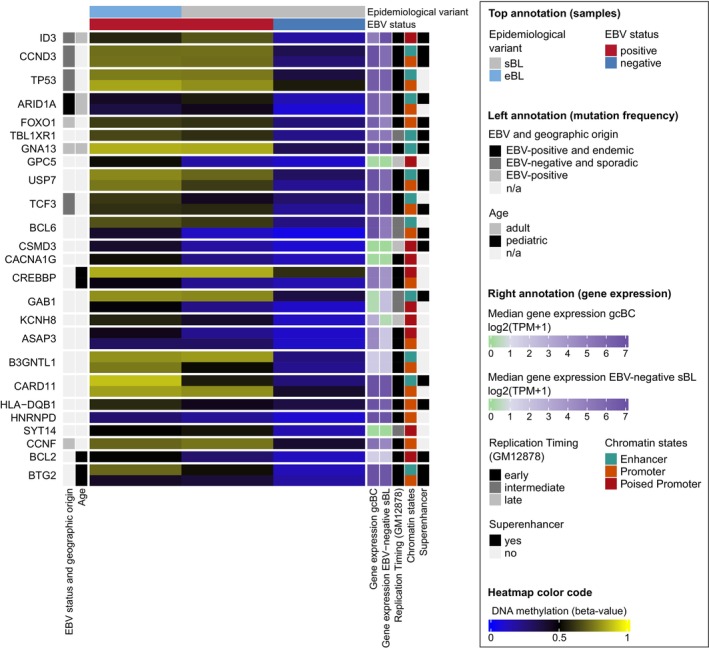
Heatmap of DNA methylation levels in regulatory regions of genes recurrently mutated in Burkitt lymphoma (BL). Heatmap displaying significant differentially methylated CpGs of EBV‐positive BL compared to EBV‐negative BL (adjusted *p* < 0.01, |Δ*β*| > 0.2) within regulatory regions (promoter, enhancer) for recurrently mutated genes in BL. Median DNA methylation for the groups EBV‐positive sBL (*n* = 6) and eBL (*n* = 19), as well as EBV‐negative sBL (*n* = 33), were calculated. In addition, median gene expression for germinal center B cells (gcBCs, *n* = 5) and EBV‐negative sBLs (*n* = 21) are included as annotation bars on the right side, showing that four genes (*CACNA1G*, *CSMD3*, *GPC5*, and *SYT14*) were not expressed in either gcBCs or sBLs. Genes are ordered according to the mutational frequency in BLs based on the findings by López et al. [4], with the gene with the highest frequency placed at the top. Gene annotations reflect multiple mutational frequency parameters: Higher frequencies based on EBV status and geographic origin [8, 9] and patient age. Additional annotations indicate replication timing in the GM12878 cell line (ENCODE Repli‐seq data), chromatin states defined in gcBCs, and superenhancers the selected CpGs are located in [36].

To further explore the potential biological significance of the extensive DNA hypermethylation, we filtered for DMCs (adj. *p* < 0.01, |Δ*β*| > 0.2; 34 361 CpGs) within regulatory regions (promoters, enhancers) for genes known to be recurrently mutated in BLs [4]. Moreover, we assessed the expression levels of these genes in gcBCs and EBV‐negative sBLs (Figure 3). We observed that EBV‐positive BLs exhibited for most of the genes (e.g., *CCND3*, *GNA13*, *TP53*, and *USP7*) higher DNAme levels compared to EBV‐negative BLs. This holds particularly true for driver genes in which a lower frequency of mutations has been previously described in EBV‐positive versus EBV‐negative BLs [8, 9]. Further, we observed differences in DNAme levels between EBV‐positive sBLs and eBLs in several genes, including *BCL6*, *BTG2*, *CARD11*, and *GPC5*. In line with previous research, the increased DNAme could potentially compensate for the lower frequency of mutations in driver genes previously observed in EBV‐positive BLs [8, 9].

## 4 Discussion

In the present study, we provide insights into the epigenetic landscape of BL from different geographic origins and with different infection burdens. By profiling 96 BL cases, we demonstrate that DNAme‐based clustering of BL is primarily driven by EBV status, leading to the identification of distinct epigenetic subgroups within BL associated with particular epidemiological and biological features. Moreover, a detailed analysis of CpGs showed differential DNAme between subgroups, which uncovered potential pathways and genes linked to BL pathogenesis. Finally, we provide further evidence that DNAme might compensate for the lower mutation frequency of driver genes in EBV‐positive as compared to EBV‐negative BL.

Our findings show that BL cases segregate into two major DNAme clusters, predominantly differing by EBV status, which is consistent with other EBV‐associated cancers, such as gastric and nasopharyngeal carcinomas [40]. Furthermore, the EBV‐positive cases formed two hypermethylated subclusters (BL‐mC1 and BL‐mC2). In comparison, EBV‐negative cases also formed two hypomethylated subclusters (BL‐mC3 and BL‐mC4). This extensive DNA hypermethylation in EBV‐positive BLs accompanies increased epigenetic age and proliferation history, likely reflecting the GC reaction history of tumor precursors.

Interestingly, two EBV‐positive cases clustered with EBV‐negative cases (BL‐mC4), raising the possibility that EBV may act as a bystander rather than a driver event in these cases. Moreover, we show that EBV‐transformed lymphoblastoid B‐cell lines (LCLs) exhibit lower DNAme levels for BL‐M1 and BL‐M2 and form a separate cluster from BL within a UMAP. Differences in EBV latency phases between LCL and EBV‐positive BL might explain these differences in DNAme [41]. Nevertheless, considering the described properties of the CpGs in the modules BL‐M1 and BL‐M2, the differences between EBV‐positive LCLs and BL could also reflect epigenetic traces of B‐cell differentiation, proliferation, and, presumably, the number of passages through the GC. These observations underscore the need for further investigation into the functional consequences of EBV infection on DNAme in benign and malignant B cells.

We identified two subclusters of EBV‐positive BL (BL‐mC1 and BL‐mC2). Therefore, despite EBV positivity representing the main factor segregating the DNAme subgroups within the BL, some additional heterogeneity exists in the DNAme patterns. Remarkably, BL‐mC1 predominantly comprises eBL cases, and BL‐mC2 is enriched for sBL cases. This segregation according to geographic origin suggests that the epidemiologic subtyping continues to hold some biologic information. In line with this, supervised analysis of EBV‐positive sBL versus eBL identified a small set of 520 DMCs. Notably, these included CpGs in a poised‐promoter region linked to the *SOX11* gene and a superenhancer region at the *RUNX1* locus, that is, two genes previously linked to EBV infection in BL and B cells, respectively [38, 39]. We cannot rule out that technical differences in sampling, population differences, or other genetic, environmental, or viral confounders cause these differences, though we have thoroughly controlled for that. Also, these differences might again be linked to GC passage as the differentially methylated CpGs are enriched for SUZ12 binding sites. In contrast, our study is not able to confirm or exclude previous exposure to malaria as a putative cause of the DNAme differences observed in the BL from Africa. At least in splenomegaly samples associated with malaria, the DNA pattern resembled that of normal lymphocyte populations rather than that of BL. Nevertheless, children with BL are exposed to hundreds of *P. falciparum* infections prior to developing BL [17]. *P. falciparum* infection may directly affect the DNA methylation states of certain immune cells in children exposed to malaria [42] or indirectly by increasing the lytic reactivation of EBV infection [43], making it difficult to disentangle the effects of EBV and *P. falciparum* from each other in BL cases arising from malaria endemic areas.

The sets of CpGs identified in the unsupervised and supervised analyses of both primary BL samples and BL‐derived cell lines provide insights into the epigenomic processes and regulatory pathways associated with EBV status. The CpGs mainly hypermethylated in EBV‐positive BLs are predominantly localized in CpG islands and poised promoter regions and are enriched for SUZ12 and EZH2 binding sites. This implicates a role of the epigenetic modifier PRC2, as it typically occurs during the GC reaction [44]. Conversely, the hypomethylated CpGs in EBV‐negative BLs reflect potential epigenetic deregulation of genes involved in B‐cell receptor signaling and FOXO pathways, both well known to be involved in BL lymphomagenesis [45, 46].

The extensive DNA hypermethylation in EBV‐positive BLs compared to EBV‐negative cases covered regulatory regions of genes frequently mutated in BL [4], such as *CCND3*, *GNA13*, *TP53*, and *USP7*, as well as many SEs [37]. This observation aligns with the hypothesis that DNA hypermethylation may compensate for the lower mutational burden in EBV‐positive BLs, potentially silencing tumor suppressor genes or modulating oncogenic pathways [8, 13].

In conclusion, our findings on DNAme patterns in BL underline previous molecular studies that suggest that the EBV status, rather than the geographic origin or immunological status of cases, is the main distinctive feature of BL subtypes [8, 9, 10, 47, 48, 49]. The strong “hypermethylator phenotype” of EBV‐positive BL shows features similar to (repetitive) GC‐passaging. This is in line with the striking genome‐wide increase in aberrant somatic hypermutation in EBV‐positive as compared to EBV‐negative BL reported by Grande et al. using genomic sequencing [8]. This finding is also in agreement with the observation that a substantial fraction of the *IG*::*MYC* fusions occurs by aberrant somatic hypermutation in EBV‐positive BL. Somatic hypermutation is a process assigned to the GC dark zone cells through which the tumor cell (precursor) must have traveled during the GC passage. In contrast, in EBV‐negative BL the pathogenesis of the *IG*::*MYC* fusion is based mostly on illegitimate class switch recombination, a process assigned to pre‐GC or GC light zone cells in line with a probable (pre‐)centroblast as the cell of origin [4, 6, 47, 50]. Overall, our findings underscore the critical role of EBV in shaping the DNAme landscape of BL.

## Author Contributions

A.R., G.O., L.T., B.B., N.O., T.O., S.L., L.L., K.B., A.B., W.W., M.R., J.N., S.M.M., M.J.B., J.T.N., G.T., and W.K. provided tumor samples and clinical data. M.H., M.S., and W.K. stained and reviewed cryomaterial, prepared, and performed quality control. W.K. and M.H. coordinated the extraction of analytes. S.E., C.L., S.B., and R.S. performed and evaluated FISH studies. R.K. provided normal B‐cell samples. O.A., P.L., C.V., and R.S. designed and coordinated the DNA methylation study. C.L., R.W., O.A., and J.K. collected and interpreted experimental data. S.G., M.K., M.L., M.R., D.H., H.K., S.H., S.B., and C.V. performed bioinformatics and biometrics analyses and provided results of bioinformatics analyses. O.A., B.R., and P.L. coordinated WP7 of the ICGC MMML‐Seq project, conducting the DNA methylation analyses. S.G., C.V., S.M.M., and R.S. interpreted data and wrote the manuscript. All authors read and approved the final manuscript.

## Conflicts of Interest

M.J.B. is currently an employee of Swedish Orphan Biovitrum A.B. The other authors declare no conflicts of interest.

## Supporting information


Table S1.

Data S1.

Figure S1.

## Data Availability

DNA methylome data produced in this study are available at GEO under the accession numbers GSE286028 and GSE286029.

## References

[1] R. Alaggio , C. Amador , I. Anagnostopoulos , et al., “The 5th Edition of the World Health Organization Classification of Haematolymphoid Tumours: Lymphoid Neoplasms,” Leukemia 36, no. 7 (2022): 1720–1748, 10.1038/s41375-022-01620-2. 35732829 PMC9214472

[2] E. G. Boerma , R. Siebert , P. M. Kluin , and M. Baudis , “Translocations Involving 8q24 in Burkitt Lymphoma and Other Malignant Lymphomas: A Historical Review of Cytogenetics in the Light of Todays Knowledge,” Leukemia 23, no. 2 (2009): 225–234, 10.1038/leu.2008.281. 18923440

[3] S. M. Aukema , L. Theil , M. Rohde , et al., “Sequential Karyotyping in Burkitt Lymphoma Reveals a Linear Clonal Evolution With Increase in Karyotype Complexity and a High Frequency of Recurrent Secondary Aberrations,” British Journal of Haematology 170, no. 6 (2015): 814–825, 10.1111/bjh.13501. 26104998

[4] C. López , K. Kleinheinz , S. M. Aukema , et al., “Genomic and Transcriptomic Changes Complement Each Other in the Pathogenesis of Sporadic Burkitt Lymphoma,” Nature Communications 10, no. 1 (2019): 1459, 10.1038/s41467-019-08578-3. PMC6440956 30926794

[5] C. López , B. Burkhardt , J. K. C. Chan , et al., “Burkitt Lymphoma,” Nature Reviews. Disease Primers 8, no. 1 (2022): 78, 10.1038/s41572-022-00404-3. 36522349

[6] N. Thomas , K. Dreval , D. S. Gerhard , et al., “Genetic Subgroups Inform on Pathobiology in Adult and Pediatric Burkitt Lymphoma,” Blood 141, no. 8 (2023): 904–916, 10.1182/blood.2022016534. 36201743 PMC10023728

[7] S. H. Swerdlow , E. Campo , N. L. Harris , et al., WHO Classification of Tumours of Haematopoietic and Lymphoid Tissues, 4th ed. (International Agency for Research on Cancer, 2017).

[8] B. M. Grande , D. S. Gerhard , A. Jiang , et al., “Genome‐Wide Discovery of Somatic Coding and Noncoding Mutations in Pediatric Endemic and Sporadic Burkitt Lymphoma,” Blood 133, no. 12 (2019): 1313–1324, 10.1182/blood-2018-09-871418. 30617194 PMC6428665

[9] F. Abate , M. R. Ambrosio , L. Mundo , et al., “Distinct Viral and Mutational Spectrum of Endemic Burkitt Lymphoma,” PLoS Pathogens 11, no. 10 (2015): e1005158, 10.1371/journal.ppat.1005158. 26468873 PMC4607508

[10] J. Richter , K. John , A. M. Staiger , et al., “Epstein–Barr Virus Status of Sporadic Burkitt Lymphoma Is Associated With Patient Age and Mutational Features,” British Journal of Haematology 196, no. 3 (2022): 681–689, 10.1111/bjh.17874. 34617271

[11] B. Burkhardt , U. Michgehl , J. Rohde , et al., “Clinical Relevance of Molecular Characteristics in Burkitt Lymphoma Differs According to Age,” Nature Communications 13, no. 1 (2022): 3881, 10.1038/s41467-022-31355-8. PMC9259584 35794096

[12] H. Kretzmer , S. H. Bernhart , W. Wang , et al., “DNA Methylome Analysis in Burkitt and Follicular Lymphomas Identifies Differentially Methylated Regions Linked to Somatic Mutation and Transcriptional Control,” Nature Genetics 47, no. 11 (2015): 1316–1325, 10.1038/ng.3413. 26437030 PMC5444523

[13] H. Hernandez‐Vargas , H. Gruffat , M. P. Cros , et al., “Viral Driven Epigenetic Events Alter the Expression of Cancer‐Related Genes in Epstein–Barr‐Virus Naturally Infected Burkitt Lymphoma Cell Lines,” Scientific Reports 7, no. 1 (2017): 5852, 10.1038/s41598-017-05713-2. 28724958 PMC5517637

[14] P. Aka , M. C. Vila , A. Jariwala , et al., “Endemic Burkitt Lymphoma Is Associated With Strength and Diversity of *Plasmodium falciparum* Malaria Stage‐Specific Antigen Antibody Response,” Blood 122, no. 5 (2013): 629–635, 10.1182/blood-2012-12-475665. 23645841 PMC3731925

[15] K. A. Lombardo , D. G. Coffey , A. J. Morales , et al., “High‐Throughput Sequencing of the B‐Cell Receptor in African Burkitt Lymphoma Reveals Clues to Pathogenesis,” Blood Advances 1, no. 9 (2017): 535–544, 10.1182/bloodadvances.2016000794. 29296973 PMC5728594

[16] B. Shiramizu , F. Barriga , J. Neequaye , et al., “Patterns of Chromosomal Breakpoint Locations in Burkitt's Lymphoma: Relevance to Geography and Epstein–Barr Virus Association,” Blood 77, no. 7 (1991): 1516–1526. 1849033

[17] K. Broen , J. Dickens , R. Trangucci , et al., “Burkitt Lymphoma Risk Shows Geographic and Temporal Associations With *Plasmodium falciparum* Infections in Uganda, Tanzania, and Kenya,” Proceedings of the National Academy of Sciences of the United States of America 120, no. 2 (2023): e2211055120, 10.1073/pnas.2211055120. 36595676 PMC9926229

[18] T. I. Lee , R. G. Jenner , L. A. Boyer , et al., “Control of Developmental Regulators by Polycomb in Human Embryonic Stem Cells,” Cell 125, no. 2 (2006): 301–313, 10.1016/j.cell.2006.02.043. 16630818 PMC3773330

[19] M. Kulis , A. Merkel , S. Heath , et al., “Whole‐Genome Fingerprint of the DNA Methylome During Human B Cell Differentiation,” Nature Genetics 47, no. 7 (2015): 746–756, 10.1038/ng.3291. 26053498 PMC5444519

[20] C. C. Oakes , M. Seifert , Y. Assenov , et al., “DNA Methylation Dynamics During B Cell Maturation Underlie a Continuum of Disease Phenotypes in Chronic Lymphocytic Leukemia,” Nature Genetics 48, no. 3 (2016): 253–264, 10.1038/ng.3488. 26780610 PMC4963005

[21] A. K. Bergmann , V. Fataccioli , G. Castellano , et al., “DNA Methylation Profiling of Hepatosplenic T‐Cell Lymphoma,” Haematologica 104, no. 3 (2019): e104–e107, 10.3324/haematol.2018.196196. 30337361 PMC6395348

[22] A. Garcia‐Gomez , T. Li , M. Kerick , et al., “TET2‐ and TDG‐Mediated Changes Are Required for the Acquisition of Distinct Histone Modifications in Divergent Terminal Differentiation of Myeloid Cells,” Nucleic Acids Research 45, no. 17 (2017): 10002–10017, 10.1093/nar/gkx666. 28973458 PMC5622316

[23] L. T. Husquin , M. Rotival , M. Fagny , et al., “Exploring the Genetic Basis of Human Population Differences in DNA Methylation and Their Causal Impact on Immune Gene Regulation,” Genome Biology 19, no. 1 (2018): 222, 10.1186/s13059-018-1601-3. 30563547 PMC6299574

[24] E. M. Kennedy , G. N. Goehring , M. H. Nichols , et al., “An Integrated ‐Omics Analysis of the Epigenetic Landscape of Gene Expression in Human Blood Cells,” BMC Genomics 19, no. 1 (2018): 476, 10.1186/s12864-018-4842-3. 29914364 PMC6006777

[25] R. M. Rodriguez , B. Suarez‐Alvarez , D. Mosén‐Ansorena , et al., “Regulation of the Transcriptional Program by DNA Methylation During Human αβ T‐Cell Development,” Nucleic Acids Research 43, no. 2 (2015): 760–774, 10.1093/nar/gku1340. 25539926 PMC4333391

[26] R. Vento‐Tormo , C. Company , J. Rodríguez‐Ubreva , et al., “IL‐4 Orchestrates STAT6‐Mediated DNA Demethylation Leading to Dendritic Cell Differentiation,” Genome Biology 17, no. 1 (2016): 4, 10.1186/s13059-015-0863-2. 26758199 PMC4711003

[27] M. J. Aryee , A. E. Jaffe , H. Corrada‐Bravo , et al., “Minfi: A Flexible and Comprehensive Bioconductor Package for the Analysis of Infinium DNA Methylation Microarrays,” Bioinformatics 30, no. 10 (2014): 1363–1369, 10.1093/bioinformatics/btu049. 24478339 PMC4016708

[28] S. H. Bernhart , H. Kretzmer , L. M. Holdt , et al., “Changes of Bivalent Chromatin Coincide With Increased Expression of Developmental Genes in Cancer,” Scientific Reports 6, no. 1 (2016): 37393, 10.1038/srep37393. 27876760 PMC5120258

[29] M. Duran‐Ferrer , G. Clot , F. Nadeu , et al., “The Proliferative History Shapes the DNA Methylome of B‐Cell Tumors and Predicts Clinical Outcome,” Nature Cancer 1, no. 11 (2020): 1066–1081, 10.1038/s43018-020-00131-2. 34079956 PMC8168619

[30] D. Pelegí‐Sisó , P. De Prado , J. Ronkainen , M. Bustamante , and J. R. González , “Methylclock: A Bioconductor Package to Estimate DNA Methylation Age,” Bioinformatics 37, no. 12 (2021): 1759–1760, 10.1093/bioinformatics/btaa825. 32960939

[31] Y. Qin , H. Feng , M. Chen , H. Wu , and X. Zheng , “InfiniumPurify: An R Package for Estimating and Accounting for Tumor Purity in Cancer Methylation Research,” Genes & Diseases 5, no. 1 (2018): 43–45, 10.1016/j.gendis.2018.02.003. 30258934 PMC6147081

[32] P. D. Johann , N. Jäger , S. M. Pfister , and M. Sill , “RF_Purify: A Novel Tool for Comprehensive Analysis of Tumor‐Purity in Methylation Array Data Based on Random Forest Regression,” BMC Bioinformatics 20, no. 1 (2019): 428, 10.1186/s12859-019-3014-z. 31419933 PMC6697926

[33] A. E. Jaffe , “FlowSorted.Blood.450k: Illumina HumanMethylation Data on Sorted Blood Cell Populations,” 2017, R Package Version 1.44.0, 10.18129/B9.BIOC.FLOWSORTED.BLOOD.450K.

[34] Z. Gu , M. Schlesner , and D. Hübschmann , “Cola: An R/Bioconductor Package for Consensus Partitioning Through a General Framework,” Nucleic Acids Research 49, no. 3 (2021): e15, 10.1093/nar/gkaa1146. 33275159 PMC7897501

[35] M. E. Ritchie , B. Phipson , D. Wu , et al., “Limma Powers Differential Expression Analyses for RNA‐Sequencing and Microarray Studies,” Nucleic Acids Research 43, no. 7 (2015): e47, 10.1093/nar/gkv007. 25605792 PMC4402510

[36] Z. Gu , “Complex Heatmap Visualization,” iMeta 1, no. 3 (2022): e43, 10.1002/imt2.43. 38868715 PMC10989952

[37] E. Bal , R. Kumar , M. Hadigol , et al., “Super‐Enhancer Hypermutation Alters Oncogene Expression in B Cell Lymphoma,” Nature 607, no. 7920 (2022): 808–815, 10.1038/s41586-022-04906-8. 35794478 PMC9583699

[38] G. Brady , H. J. Whiteman , L. C. Spender , and P. J. Farrell , “Downregulation of RUNX1 by RUNX3 Requires the RUNX3 VWRPY Sequence and Is Essential for Epstein–Barr Virus‐Driven B‐Cell Proliferation,” Journal of Virology 83, no. 13 (2009): 6909–6916, 10.1128/JVI.00216-09. 19403666 PMC2698531

[39] M. Sureda‐Gómez , I. Iaccarino , A. De Bolòs , et al., “SOX11 Expression Is Restricted to EBV‐Negative Burkitt Lymphoma and Is Associated With Molecular Genetic Features,” Blood 144, no. 2 (2024): 187–200, 10.1182/blood.2023023242. 38620074

[40] L. J. Stanland and M. A. Luftig , “The Role of EBV‐Induced Hypermethylation in Gastric Cancer Tumorigenesis,” Viruses 12, no. 11 (2020): 1222, 10.3390/v12111222. 33126718 PMC7693998

[41] G. Niedobitek , A. Agathanggelou , M. Rowe , et al., “Heterogeneous Expression of Epstein–Barr Virus Latent Proteins in Endemic Burkitt's Lymphoma,” Blood 86, no. 2 (1995): 659–665, 10.1182/blood.V86.2.659.bloodjournal862659. 7605996

[42] D. Almojil , A. Diawara , I. Soulama , et al., “Impact of *Plasmodium falciparum* Infection on DNA Methylation of Circulating Immune Cells,” Frontiers in Genetics 14 (2023): 1197933, 10.3389/fgene.2023.1197933. 37470040 PMC10352500

[43] H. Watier , C. Auriault , and A. Capron , “Does Epstein–Barr Virus Infection Confer Selective Advantage to Malaria‐Infected Children?,” Lancet 341, no. 8845 (1993): 612–613, 10.1016/0140-6736(93)90364-m. 8094840

[44] I. Velichutina , R. Shaknovich , H. Geng , et al., “EZH2‐Mediated Epigenetic Silencing in Germinal Center B Cells Contributes to Proliferation and Lymphomagenesis,” Blood 116, no. 24 (2010): 5247–5255, 10.1182/blood-2010-04-280149. 20736451 PMC3012542

[45] J. Corso , K. T. Pan , R. Walter , et al., “Elucidation of Tonic and Activated B‐Cell Receptor Signaling in Burkitt's Lymphoma Provides Insights Into Regulation of Cell Survival,” Proceedings of the National Academy of Sciences of the United States of America 113, no. 20 (2016): 5688–5693, 10.1073/pnas.1601053113. 27155012 PMC4878517

[46] E. Kabrani , V. T. Chu , E. Tasouri , et al., “Nuclear FOXO1 Promotes Lymphomagenesis in Germinal Center B Cells,” Blood 132, no. 25 (2018): 2670–2683, 10.1182/blood-2018-06-856203. 30333121

[47] C. Bellan , S. Lazzi , M. Hummel , et al., “Immunoglobulin Gene Analysis Reveals 2 Distinct Cells of Origin for EBV‐Positive and EBV‐Negative Burkitt Lymphomas,” Blood 106, no. 3 (2005): 1031–1036, 10.1182/blood-2005-01-0168. 15840698

[48] Y. Kaymaz , C. I. Oduor , H. Yu , et al., “Comprehensive Transcriptome and Mutational Profiling of Endemic Burkitt Lymphoma Reveals EBV Type‐Specific Differences,” Molecular Cancer Research 15, no. 5 (2017): 563–576, 10.1158/1541-7786.MCR-16-0305. 28465297 PMC5471630

[49] L. Leoncini , “Epstein–Barr Virus Positivity as a Defining Pathogenetic Feature of Burkitt Lymphoma Subtypes,” British Journal of Haematology 196, no. 3 (2022): 468–470, 10.1111/bjh.17922. 34725813 PMC9298118

[50] J. A. Roco , L. Mesin , S. C. Binder , et al., “Class‐Switch Recombination Occurs Infrequently in Germinal Centers,” Immunity 51, no. 2 (2019): 337–350.e7, 10.1016/j.immuni.2019.07.001. 31375460 PMC6914312

