# Supplementary material for: Subtyping Burkitt Lymphoma by DNA Methylation
Source: Genes Chromosomes Cancer. 2025 Apr 7;64(4):e70042. doi: 10.1002/gcc.70042 (PMC11974478; doi:10.1002/gcc.70042)
Supplement: Supplementary file 2 — Data S1. [file GCC-64-e70042-s001.docx]

# Supplementary Information

## Full list of members of the ICGC MMML-seq

*Coordination (C1):* Reiner Siebert^1,2^, Susanne Wagner^2^, Andrea Haake^2^, Julia Richter^2,3^, Gesine Richter^2^

*Data Center (C2):* Roland Eils^4,5^, Chris Lawerenz^4^, Jürgen Eils^4^, Jules Kerssemakers^4^, Christina Jaeger-Schmidt^4^, Ingrid Scholz^4^

*Clinical Centers (WP1):* Anke K. Bergmann^2, 6^*,* Christoph Borst^7^, Birgit Burkhardt^8,9^, Alexander Claviez^6^, Martin Dreyling^10^, Sonja Eberth^11^, Hermann Einsele^12^, Norbert Frickhofen^13^, Siegfried Haas^7^, Martin-Leo Hansmann^14^, Dennis Karsch^15^, Michael Kneba^15^, Jasmin Lisfeld^9^, Luisa Mantovani-Löffler^16^, Marius Rohde^9^, German Ott^17^, Christina Stadler^11^, Peter Staib^18^, Stephan Stilgenbauer^19^, Lorenz Trümper^11^, Thorsten Zenz^20^

*Normal Cells (WPN):* Martin-Leo Hansmann^14^, Dieter Kube^11^, Ralf Küppers^21^, Marc Weniger^21^

*Pathology and Analyte Preparation (WP2-3):* Michael Hummel^22^, Wolfram Klapper^3^, Ulrike Kostezka^23^, Dido Lenze^22^, Peter Möller^24^, Andreas Rosenwald^25^, German Ott^17^, Monika Szczepanowski^3^

*Sequencing and genomics (WP4-7):* Ole Ammerpohl^1,2^, Sietse M. Aukema^2,3^, Vera Binder^26^, Arndt Borkhardt^26^, Andrea Haake^2^, Jessica I. Hoell^26^; Ellen Leich^25^, Peter Lichter^27^, Cristina López^1,2^, Inga Nagel^2^, Jordan Pischimariov^25^, Bernhard Radlwimmer^27^, Julia Richter^2,3^, Philip Rosenstiel^28^, Andreas Rosenwald^25^, Markus Schilhabel^28^, Stefan Schreiber^29^, Inga Vater^2^, Rabea Wagener^1,2^, Reiner Siebert^1,2^

*Bioinformatics (WP8-9):* Stephan H. Bernhart^30-32,^ Hans Binder^30,31^, Benedikt Brors^33^, Gero Doose^30-32^, Roland Eils^4,5^, Steve Hoffmann^30-32^, Lydia Hopp^30^, Daniel Hübschmann^4,5,34^, Kortine Kleinheinz^4,5^, Helene Kretzmer^30-32^, Markus Kreuz^35^, Jan Korbel^36^, David Langenberger^30-32^, Markus Loeffler^35^, Maciej Rosolowski^35^, Matthias Schlesner^4,37^, Peter F. Stadler^30-32,38-40^, Stephanie Sungalee^36^

^1^Institute of Human Genetics, University of Ulm and University Hospital of Ulm, Ulm, Germany

^2^Institute of Human Genetics, Christian-Albrechts-University, Kiel, Germany;

^3^Hematopathology Section, Institute of Pathology, Christian-Albrechts-University, Kiel, Germany;

^4^Division of Theoretical Bioinformatics (B080), German Cancer Research Center (DKFZ), Heidelberg, Germany;

^5^Department for Bioinformatics and Functional Genomics, Institute of Pharmacy and Molecular Biotechnology and Bioquant, University of Heidelberg, Heidelberg, Germany;

^6^Department of Pediatrics, University Hospital Schleswig-Holstein, Campus Kiel, Kiel, Germany;

^7^Department of Internal Medicine/Hematology, Friedrich-Ebert-Hospital, Neumünster;

^8^University Hospital Muenster - Pediatric Hematology and Oncology, Muenster Germany;

^9^University Hospital Giessen, Pediatric Hematology and Oncology, Giessen, Germany;

^10^Department of Medicine III - Campus Grosshadern, University Hospital Munich, Munich, Germany;

^11^Department of Hematology and Oncology, Georg-August-University of Göttingen, Göttingen, Germany;

^12^University Hospital Würzburg, Department of Medicine and Poliklinik II, University of Würzburg, Würzburg;

^13^Department of Medicine III, Hematology and Oncology, Dr. Horst-Schmidt-Kliniken of Wiesbaden, Wiesbaden;

^14^Senckenberg Institute of Pathology, University of Frankfurt Medical School, Frankfurt am Main, Germany

^15^Department of Internal Medicine II: Hematology and Oncology, University Medical Centre, Campus Kiel, Kiel;

^16^Hospital of Internal Medicine II, Hematology and Oncology, St-Georg Hospital Leipzig, Leipzig, Germany;

^17^Department of Clinical Pathology, Robert-Bosch Krankenhaus, and Dr. Margarete Fischer-Bosch Institute of Clinical Pharmacology, Stuttgart, Germany;

^18^Clinic for Hematology and Oncology, St.-Antonius-Hospital, Eschweiler;

^19^Department for Internal Medicine III, University of Ulm and University Hospital of Ulm, Ulm, Germany

^20^National Centre for Tumor Disease, Heidelberg, Germany;

^21^Institute of Cell Biology (Cancer Research), University of Duisburg-Essen, Medical School, Essen, Germany;

^22^CharitéCenter for Biomedicine (CC4), Charité – University Medicine Berlin, Berlin, Germany;

^23^Comprehensive Cancer Center Ulm (CCCU), University Hospital Ulm, Ulm, Germany;

^24^Institute of Pathology, University of Ulm and University Hospital of Ulm, Ulm;

^25^Institute of Pathology, University of Wurzburg, Germany;

^26^Department of Pediatric Oncology, Hematology and Clinical Immunology, Heinrich-Heine-University, Düsseldorf, Germany;

^27^German Cancer Research Center (DKFZ), Division of Molecular Genetics, Heidelberg, 69120, Germany;

^28^Institute of Clinical Molecular Biology, Christian-Albrechts-University, Kiel, Germany;

^29^Department of General Internal Medicine, University Kiel, Kiel, Germany;

^30^Interdisciplinary Center for Bioinformatics, University of Leipzig, Leipzig, Germany;

^31^Bioinformatics Group, Department of Computer, University of Leipzig, Leipzig, Germany;

^32^Transcriptome Bioinformatics, LIFE Research Center for Civilization Diseases, University of Leipzig, Leipzig, Germany;

^33^Division of Applied Bioinformatics (G200), German Cancer Research Center (DKFZ), Heidelberg, Germany

^34^Department of Pediatric Immunology, Hematology and Oncology, University Hospital, Heidelberg, Germany

^35^Institute for Medical Informatics Statistics and Epidemiology, University of Leipzig, Leipzig, Germany;

^36^EMBL Heidelberg, Genome Biology, Heidelberg, Germany;

^37^Bioinformatics and Omics Data Analytics (B240), German Cancer Research Center (DKFZ), Heidelberg, Germany;

^38^RNomics Group, Fraunhofer Institute for Cell Therapy and Immunology IZI, Leipzig, Germany

^39^Santa Fe Institute, Santa Fe, New Mexico, United States of America

^40^Max-Planck-Institute for Mathematics in Sciences, Leipzig, Germany.

## Full list of members of the MMML

*Pathology group and analytes preparation:* Thomas F.E. Barth^1^, Heinz-Wolfram Bernd^2^, Sergio B. Cogliatti^3^, Alfred C. Feller^2^, Martin L. Hansmann^4^, Michael Hummel^5^, Wolfram Klapper^6^, Dido Lenze^5^, Peter Möller^1^, Hans-Konrad Müller-Hermelink^7^, German Ott^7^, Andreas Rosenwald^7^, Harald Stein^5^, Monika Szczepanowski^6^, Hans-Heinrich Wacker^6^.

*Genetics group:* Thomas F.E. Barth^1^, Petra Behrmann^8^, Peter Daniel^10^, Judith Dierlammm^8^, Eugenia Haralambieva^7^, Lana Harder^11^, Paul-Martin Holterhus^12^, Ralf Küppers^13^, Dieter Kube^13^, Peter Lichter^14^, Jose I. Martín-Subero^11^, Peter Möller^1^, Eva M. Murga-Peñas^9^, German Ott^7^, Christiane Pott^16^, Armin Pscherer^15^, Andreas Rosenwald^7^, Carsten Schwaenen^17^, Reiner Siebert^11^, Heiko Trautmann^16^, Martina Vockerodt^18^, Swen Wessendorf^16^.

*Bioinformatics group:* Stefan Bentink^19^, Hilmar Berger^20^, Dirk Hasenclever^20^, Markus Kreuz^20^, Markus Loeffler^20^, Maciej Rosolowski^20^, Rainer Spang^19^.

*Project coordination:* Benjamin Stürzenhofecker^14^, Lorenz Trümper^14^, Maren Wehner^14^.

*Steering committee:* Markus Loeffler^19^, Reiner Siebert^11^, Harald Stein^5^, Lorenz Trümper^14^.

^1^Institute of Pathology, University Hospital of Ulm, Ulm, Germany;

^2^Institute of Pathology, University Hospital Schleswig-Holstein Campus Lübeck, Lübeck, Germany;

^3^Institute of Pathology, Kantonsspital St. Gallen, St.Gallen, Switzerland;

^4^Institute of Pathology, University Hospital of Frankfurt, Frankfurt, Germany;

^5^Institute of Pathology, Campus Benjamin Franklin, Charité–Universitätsmedizin Berlin, Berlin, Germany;

^6^Institute of Hematopathology, University Hospital Schleswig-Holstein Campus Kiel/ Christian-Albrechts University Kiel, Kiel, Germany;

^7^Institute of Pathology, University of Würzburg, Würzburg, Germany;

^8^Cytogenetic and Molecular Diagnostics, Internal Medicine III, University Hospital of Ulm, Ulm, Germany;

^9^University Medical Center Hamburg-Eppendorf, Hamburg, Germany;

^10^Department of Hematology, Oncology and Tumor Immunology, University Medical Center Charité, Berlin, Germany;

^11^Institute of Human Genetics, University Hospital Schleswig-Holstein Campus Kiel/Christian-Albrechts University Kiel, Kiel, Germany;

^12^Division of Pediatric Endocrinology and Diabetes, Department of Pediatrics, University Hospital Schleswig-Holstein Campus Kiel / Christian-Albrechts University Kiel, Kiel, Germany;

^13^Institute for Cell Biology (Tumor Research), University of Duisburg-Essen, Essen, Germany;

^14^Department of Hematology and Oncology, Georg-August University of Göttingen, Göttingen, Germany;

^15^German Cancer Research Center (DKFZ), Heidelberg, Germany;

^16^Second Medical Department, University Hospital Schleswig-Holstein Campus Kiel/ Christian-Albrechts University Kiel, Kiel, Germany;

^17^Cytogenetic and Molecular Diagnostics, Internal Medicine III, University Hospital of Ulm, Ulm, Germany; ^18^Department of Pediatrics I, Georg-August University of Göttingen, Göttingen, Germany;

^19^Institute of Functional Genomics, University of Regensburg, Regensburg, Germany;

^20^Institute for Medical Informatics, Statistics and Epidemiology, University of Leipzig, Leipzig, Germany.

# Supplementary Methods

## Control DNA methylation data quality

Geographic differences: As the cohort encompasses European and African individuals, CpGs with an allele frequency difference > 2% were excluded from further analysis. Therefore, the 1,000 genomes phase 3 data (ftp://[ftp.1000genomes.ebi.ac.uk/vol1/ftp/phase3/](http://ftp.1000genomes.ebi.ac.uk/vol1/ftp/phase3/)) were used to calculate the allele frequency difference between the European and African population. Overall 12 million SNPs with an allele frequency > 2% were identified, overlapping with 5,648 CpGs within the set of 441,870 CpGs, after merging the datasets from Infinium HumanMethylation450 and MethylationEPIC BeadChips (Illumina Inc., San Diego, CA, USA).

Purity classifier: Since tumor samples consist of mixed cell populations, meaning that beside tumor cells various normal B-cells are included in the sample, the tumor cell content (TCC) of every sample was taken into account. The TCC, afterwards called purity, of the samples was assessed by whole genome sequencing (WGS) data from the ICGC MMML-Seq project. Therefore, for all cases for which WGS data were available the TCC was estimated based on this data as described previously.^1^. Unfortunately, not for every sample included in this study, WGS data were available. Thus, a method which allows to identify lymphoma samples with low purity was developed. This purity classifier was built on a combination of two independent analyses, shortly discussed afterwards.

In a first step we defined significant differentially methylated loci between benign B-cell (n=93) and T-cell (n=49) subpopulations, macrophages (n=9) and monocytes (n=23) in comparison to 68 germinal center-derived B-cell (gcBC) lymphomas (Burkitt lymphoma, follicular lymphoma, diffuse large B-cell lymphoma showing a TCC of more than 70% (referred to as lymphoma high TCC).^2^ A two group comparison of overall 93 benign B-cells of varying developmental stages in comparison to 68 lymphomas with high TCC was performed using settings which lead to the identification of 64 highly differentially methylated loci (σ/σmax > 0.8, q-value < 1e-20; OMICS Explorer 3.2, Qlucore, Lund, Sweden). Of note, 30 of these loci exhibited a higher DNA methylation in the lymphoma high TCC group whereas 34 loci were significantly lower methylated (Figure S1A-B). In a next step the mean DNA methylation of these 30 loci was determined for every sample. The cut-off for being of higher purity based on the DNA methylation of these 30 loci was defined using the lowest mean methylation value of the lymphoma group harboring an intermediate TCC (50-70%), which was 0.55. Samples harboring a mean DNA methylation of these 30 loci smaller than 0.55 were excluded from further analysis.

The second method is based on the DNA methylation of the poised promoter regions. Bernhart et al. recently described, that gcBC as well as naive B-cells showed at poised promoter regions a significantly lower DNA methylation in comparison to gcBC lymphomas ^3^. In the work of this group overall 14 frequently bivalent segments (FBS) were identified. Using these FBS we identified 1,058 corresponding loci on the 450k array. After calculating the mean DNA methylation of these loci, we used the overall DNA methylation of all autosomal loci on the 450k to normalize the data. Using the mean relative FBS methylation of all gcBC samples, we defined a cutoff at a relative FBS methylation of 0.55. Samples harboring a lower relative FBS methylation were excluded from further analysis.

Combining these two methods, we obtained a DNA methylation-based purity classifier, which lead to the exclusion of overall 20 BL cases (Figure S1C).

## Chromatin Mapping

The chromatin states from gcBCs described by Kretzmer et al. were used.^4^ The raw sequencing data are publically available from the European Nucleotide Archive (ENA).

## Superenhancer

Superenhancer annotations for 3,755 were obtained from Bal et al.^5^ These superenhancer were associated with 34,126 CpGs, with an average of 15 CpGs per superenhancer.

# References

1. López C, Kleinheinz K, Aukema SM, et al. Genomic and transcriptomic changes complement each other in the pathogenesis of sporadic Burkitt lymphoma. *Nat Commun*. 2019;10(1):1459. doi:10.1038/s41467-019-08578-3

2. Hübschmann D, Kleinheinz K, Wagener R, et al. Mutational mechanisms shaping the coding and noncoding genome of germinal center derived B-cell lymphomas. *Leukemia*. 2021;35(7):2002-2016. doi:10.1038/s41375-021-01251-z

3. Bernhart SH, Kretzmer H, Holdt LM, et al. Changes of bivalent chromatin coincide with increased expression of developmental genes in cancer. *Sci Rep*. 2016;6(1):37393. doi:10.1038/srep37393

4. Kretzmer H, Bernhart SH, Wang W, et al. DNA methylome analysis in Burkitt and follicular lymphomas identifies differentially methylated regions linked to somatic mutation and transcriptional control. *Nat Genet*. 2015;47(11):1316-1325. doi:10.1038/ng.3413

5. Bal E, Kumar R, Hadigol M, et al. Super-enhancer hypermutation alters oncogene expression in B cell lymphoma. *Nature*. 2022;607(7920):808-815. doi:10.1038/s41586-022-04906-8
