# Supplementary material for: Subtyping Burkitt Lymphoma by DNA Methylation
Source: Genes Chromosomes Cancer. 2025 Apr 7;64(4):e70042. doi: 10.1002/gcc.70042 (PMC11974478; doi:10.1002/gcc.70042)
Supplement: Supplementary file 3 — Figure S1. [file GCC-64-e70042-s002.pdf]

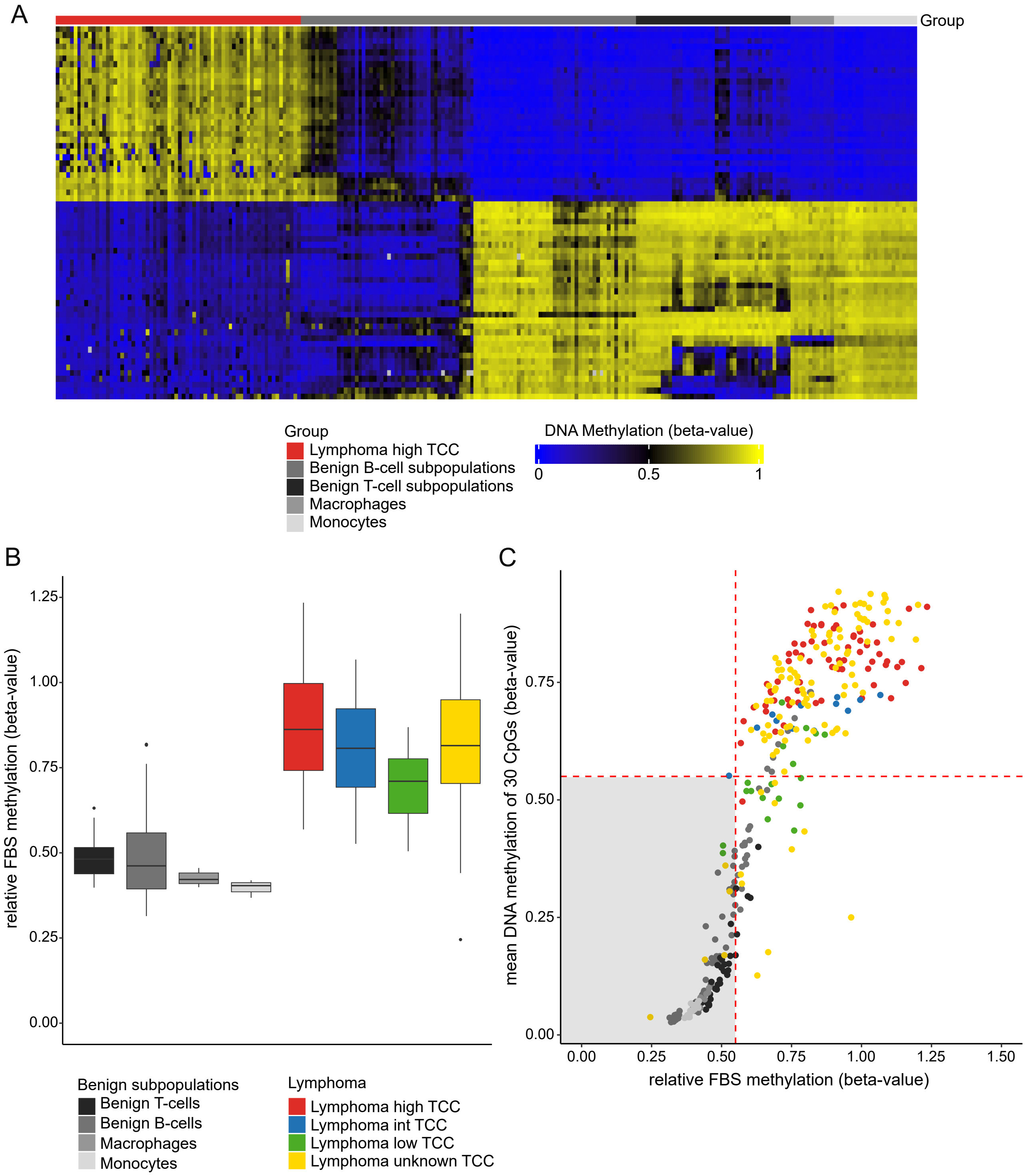

Supplementary Figure S1: DNA methylation-based purity classifier. A: Heatmap displaying the beta values of 64 highly significant differentially methylated CpGs (σ/σmax=0.8, q<=1e-20) in 93 benign B-cell populations, 49 benign T-cell populations, 23 monocytes, 9 macrophages versus 68 B-cell lymphomas (Burkitt lymphoma, diffuse large B-cell lymphoma, follicular lymphoma) with a tumor cell content (TCC) higher than 70% as estimated by whole genome sequencing. Of those 64 CpGs, 30 were hypermethylated in the B-cell lymphomas, 34 were hypomethylated. Columns encode samples and rows display CpGs. **B:** Box plot showing relative DNA methylation of CpGs within frequently bivalent segments (FBS) of benign subpopulations and B-cell lymphomas with varying TCC. The FBS segments which were described to be differentially methylated between benign B-cells and B-cell lymphomas were recently published by Bernhart et al. (28). The relative FBS DNA methylation decreases with decreasing TCC. **C:** Scatter plot combining the values of the mean DNA methylation of the 30 hypermethylated CpGs in the B-cell lymphomas with high TCC (y-axis) and the relative FBS DNA methylation (x-ayis). Cases with a mean DNA methylation for the 30 CpGs below 0.55 and a relative FBS DNA methylation below 0.55 were excluded from further analysis (grey background). Lymphoma high TCC: > 70% TCC; lymphoma int TCC: 50% < TCC < 70%; lymphoma low TCC: TCC < 30%; gcBC: germinal center B-cells. Differential DNA methylation analysis was performed with the OMICS Explorer 3.2 (Qlucore, Lund, Sweden).

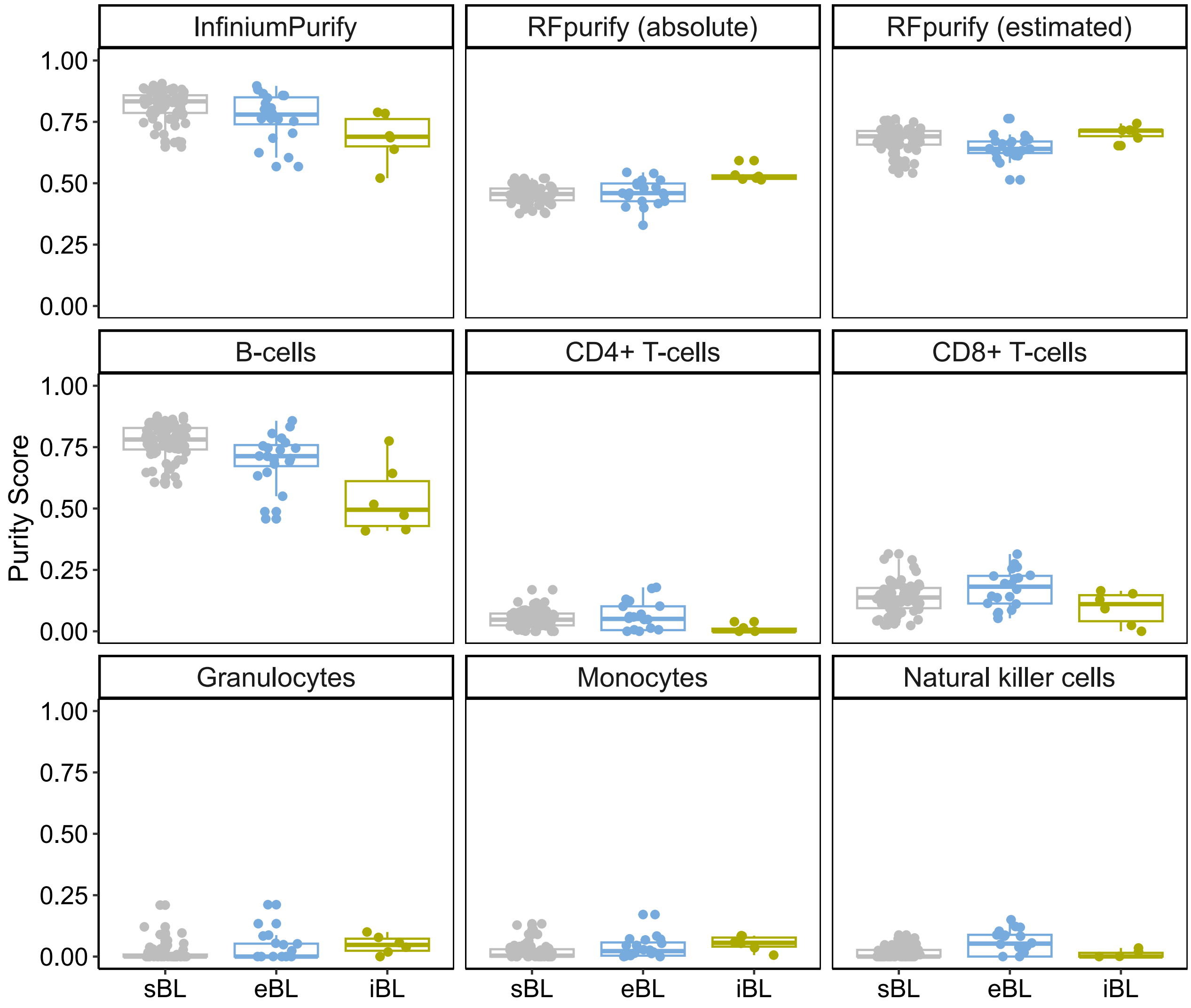

Supplementary Figure S2: DNA methylation-based calculations of cellular composition and purity scores. The box plots display various purity scores and cellular composition. sBL: sporadic Burkitt lymphoma; eBL: endemic Burkitt lymphoma; iBL: immunodeficiency-associated Burkitt lymphoma.

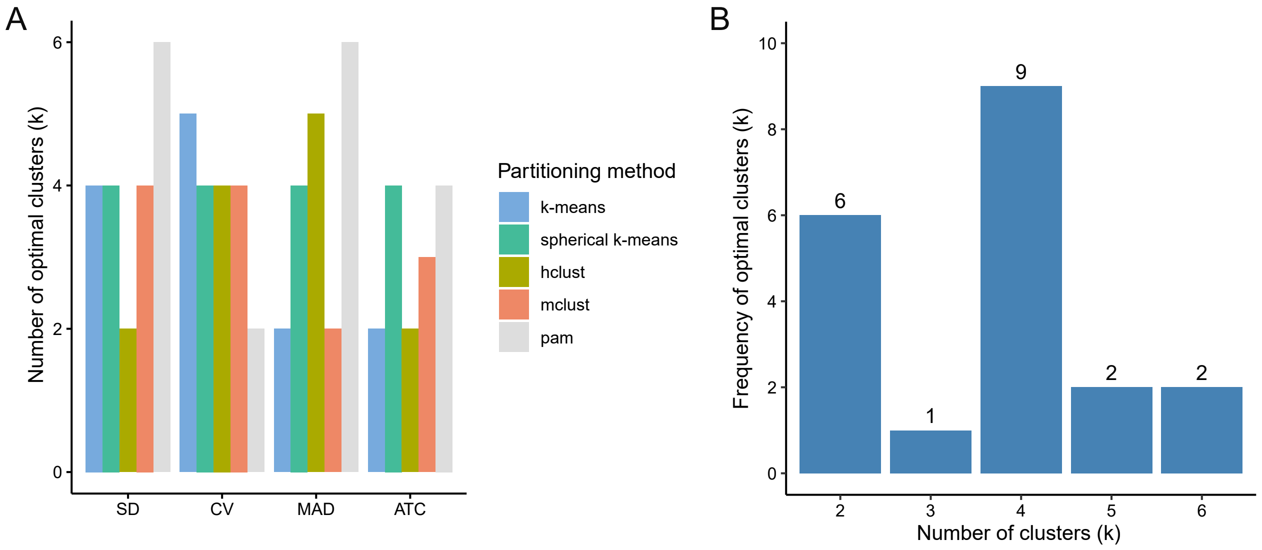

Supplementary Figure S3: Summary of optimal number of k clusters. Various consensus partitioning mehtods (k-means, spherical k-means, hclust, mclust, pam) using different CpG selection strategies (SD, CV, MAD, ATC) were applied on the 309,078 CpGs and the 96 primary Burkitt lymphoma cases using the cola package (34). **A:** Bar plot displaying the optimal number of k clusters for each of the 20 method combinations. Each bar represents a unique combination, with the height indicating the optimal k determined for that combination. **B:** Frequency distribution of optimal k clusters. The bar plot shows how often each number of clusters (k) was identified as optimal across all 20 combinations. The x-axis represents the possible k numbers, while the y-axis shows the count of combinations that yielded each k as optimal. SD: standard deviation; CV: coefficient of variance; MAD: median absolute deviation; ATC: ability to correlate to other rows; hclust: hierarchical clustering + cutree; mclust: model-based clustering; pam: partitioning around medoids. Statistics are summarized in Supplementary Table 3.

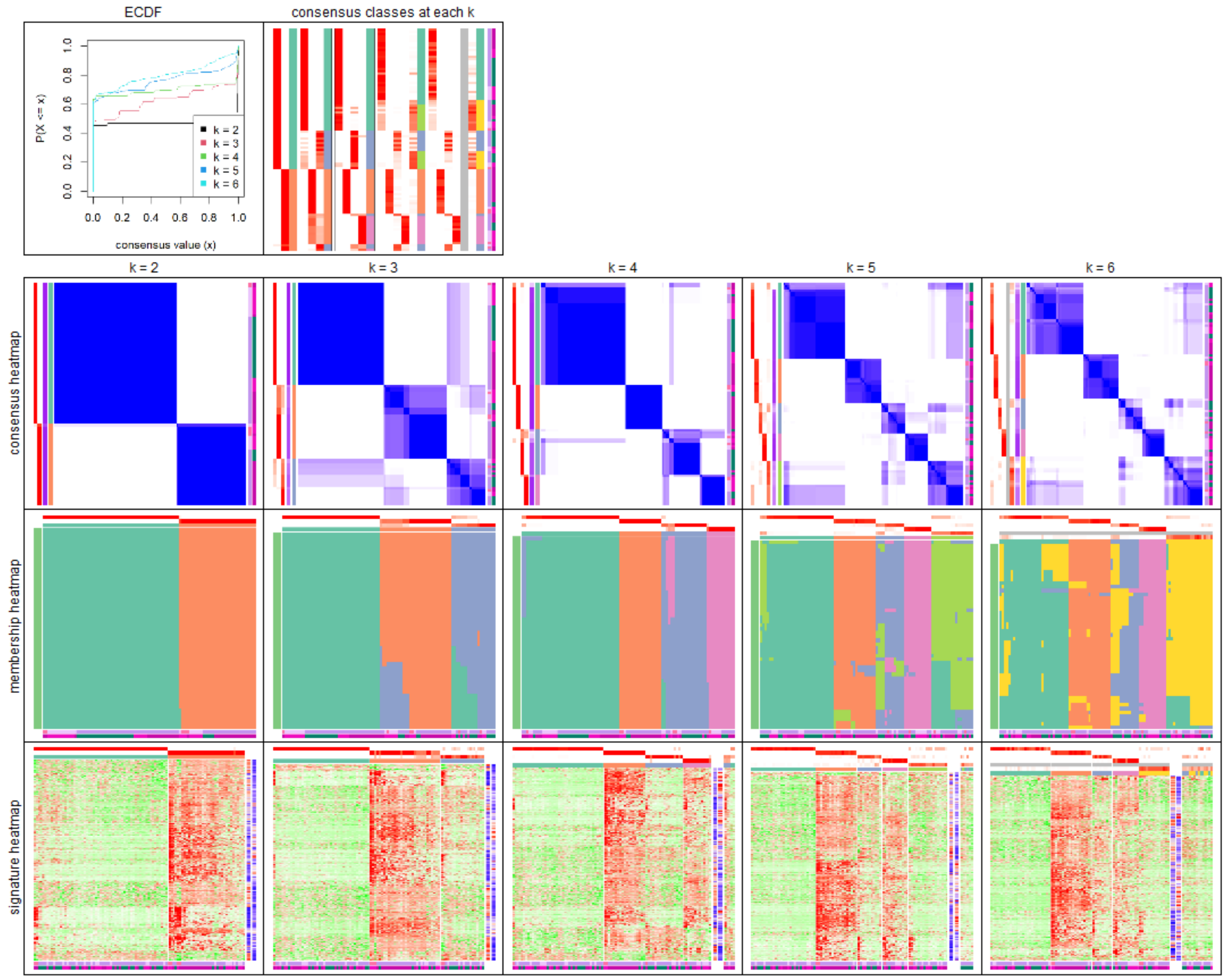

Supplementary Figure S4: Consensus clustering of DNA methylation data in Burkitt lymphoma. This plot displays the output of the collect_plot() function from the cola package (34), utilizing the standard deviation for feature selection and k-means clustering. The analysis was performed on 309,078 CpGs across 96 Burkitt lymphoma cases. The plots show the empirical cumulative distribution (ECDF) curve of the consensus matrix, the consensus datasets, the consensus heatmaps, the membership heatmaps and signature heatmaps for each k.

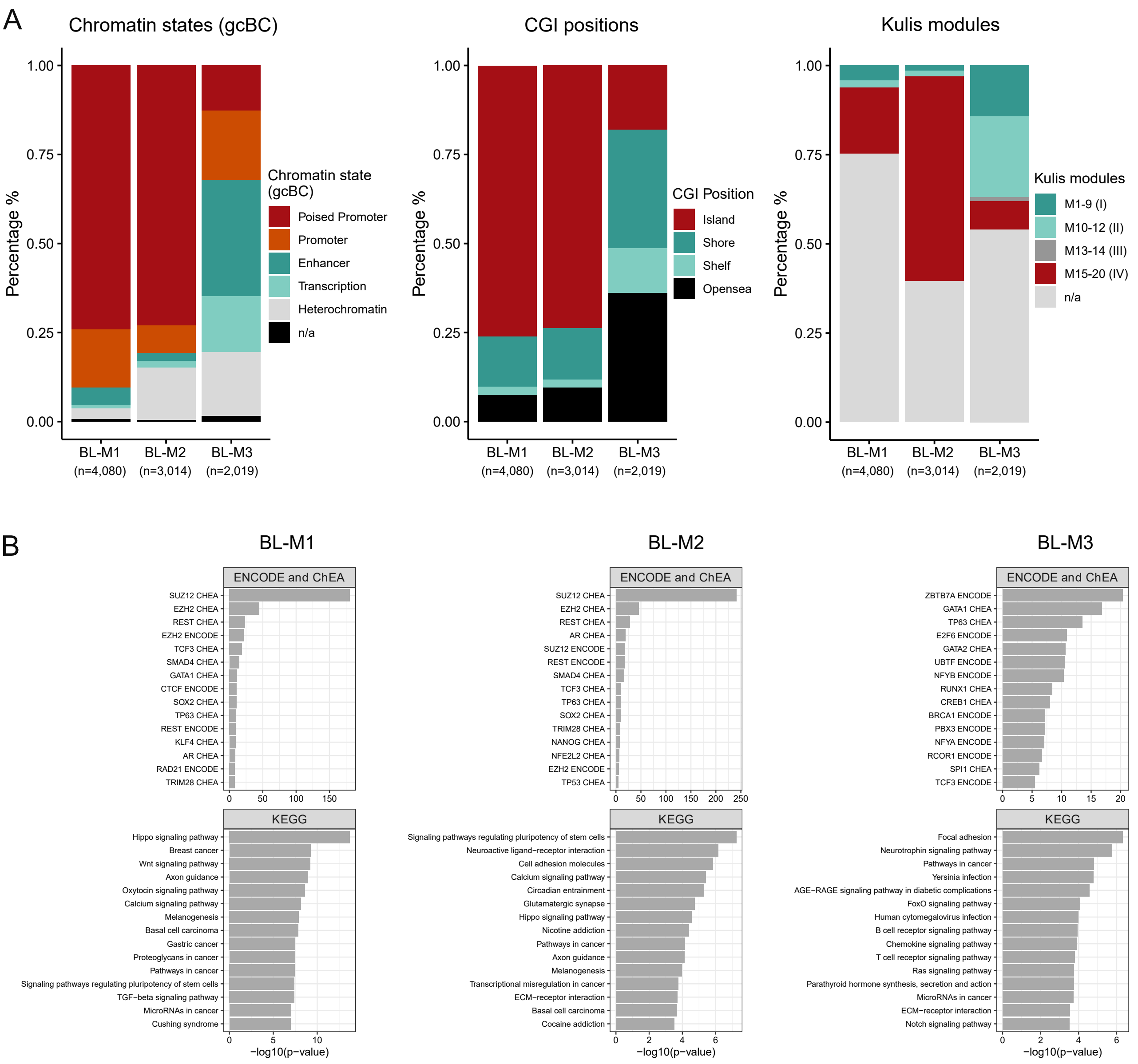

Supplementary Figure S5: Characterization of the 9,313 CpGs across BL-M1-M3. A: Bar plots display distribution of the CpGs within chromatin states defined in germinal center-derived B-cells (gcBCs) derived from Kretzmer et al. (left), their positions relative to CpG islands as annotated in the Illumina manifest (CGIs, middle) and within the dynamic modules defined by Kulis et al. (right). The Kulis modules are summarized according the defined patterns (I-IV) within the paper. Statistical comparisons are summarized in Supplementary Table 6. Shelf: ~4 Kb from islands; Shore: ~2 Kb from islands. n/a: not applicable. **B:** Transcription factor (upper panel) and pathway (lower panel) enrichment analysis of the genes associated with the modules BL-M1-M3.

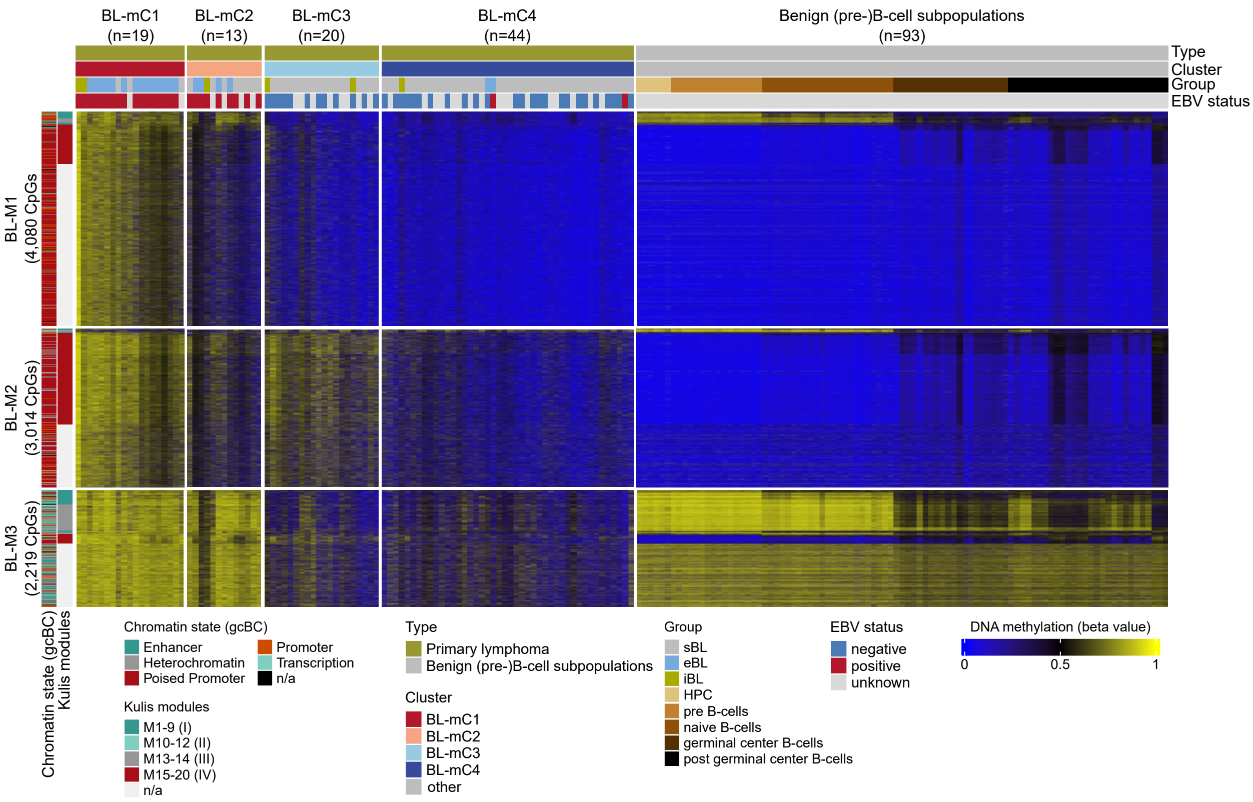

Supplementary Figure S6: DNA methylation levels of the 9,313 CpGs across non-malignant (pre-)B-cell subpopulations. Heatmap is adapted from Figure 1 and expanded to incorporate non-malignant (pre-)B-cell subpopulations (n=93). Rows represent individual CpGs, and columns represent samples. B-cell subpopulations are organized according their differentiation state. n/a: not applicable.

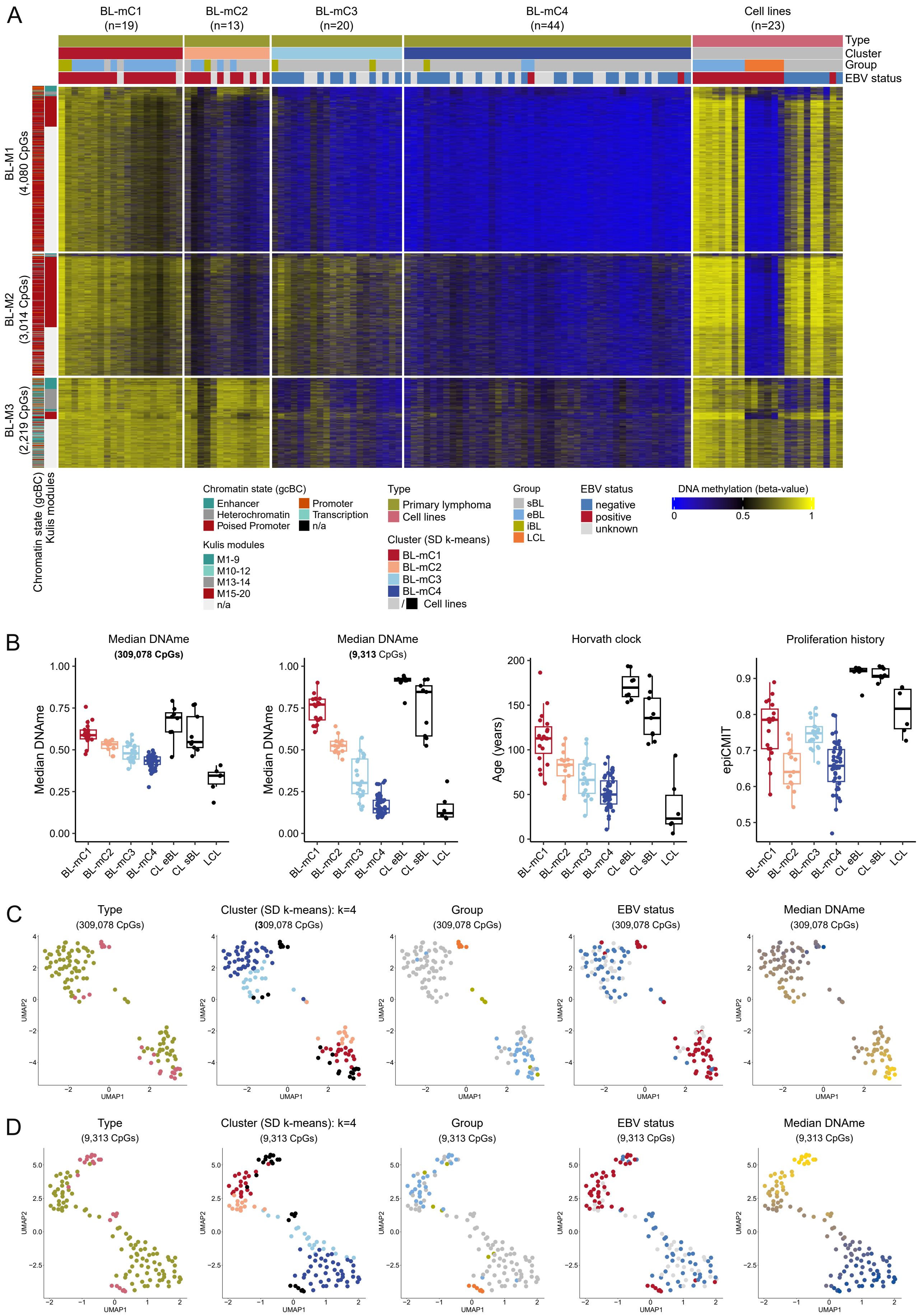

Supplementary Figure S7: Comparative DNA methylation profiling of primary Burkitt lymphoma cases with cell lines. A: Heatmap is adapted from Figure 1 and expanded to incorporate 23 cell lines (17 Burkitt lymphoma-derived cell lines [CL], 6 lymphoblastic cell lines [LCL]). Rows represent individual CpGs, and columns represent samples. **B:** Box plots illustrating key epigenetic features, including epigenetic age (Horvath clock), proliferation history (epiCMIT), and median DNA methylation levels based on the 309,078 CpGs and 9,313 CpGs. **C-D:** UMAP analysis based on the 309,078 CpGs (C) and the 9,313 CpGs (D). n/a: not applicable.

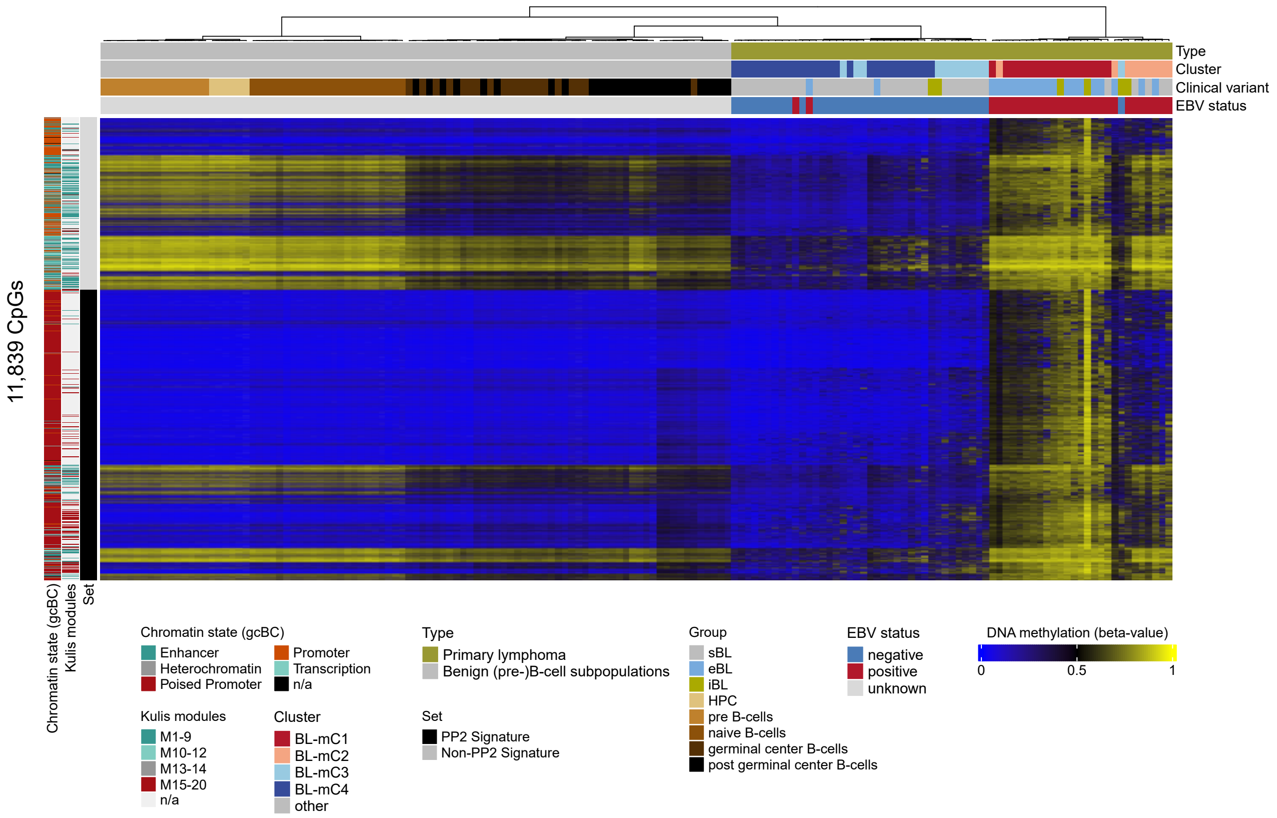

Supplementary Figure S8: DNA methylation levels of the 11,839 CpGs across non-malignant (pre-)B-cell subpopulations. Heatmap is adapted from Figure 2b and expanded to incorporate non-malignant (pre-)B-cell subpopulations (n=93). Rows represent individual CpGs, and columns represent samples. CpGs are organized according the defined signatures: PP2 Signature, Non-PP2 Signature. PP2: Poised promoter and polycomb repressive complex 2; GC: germinal center; n/a: not applicable.

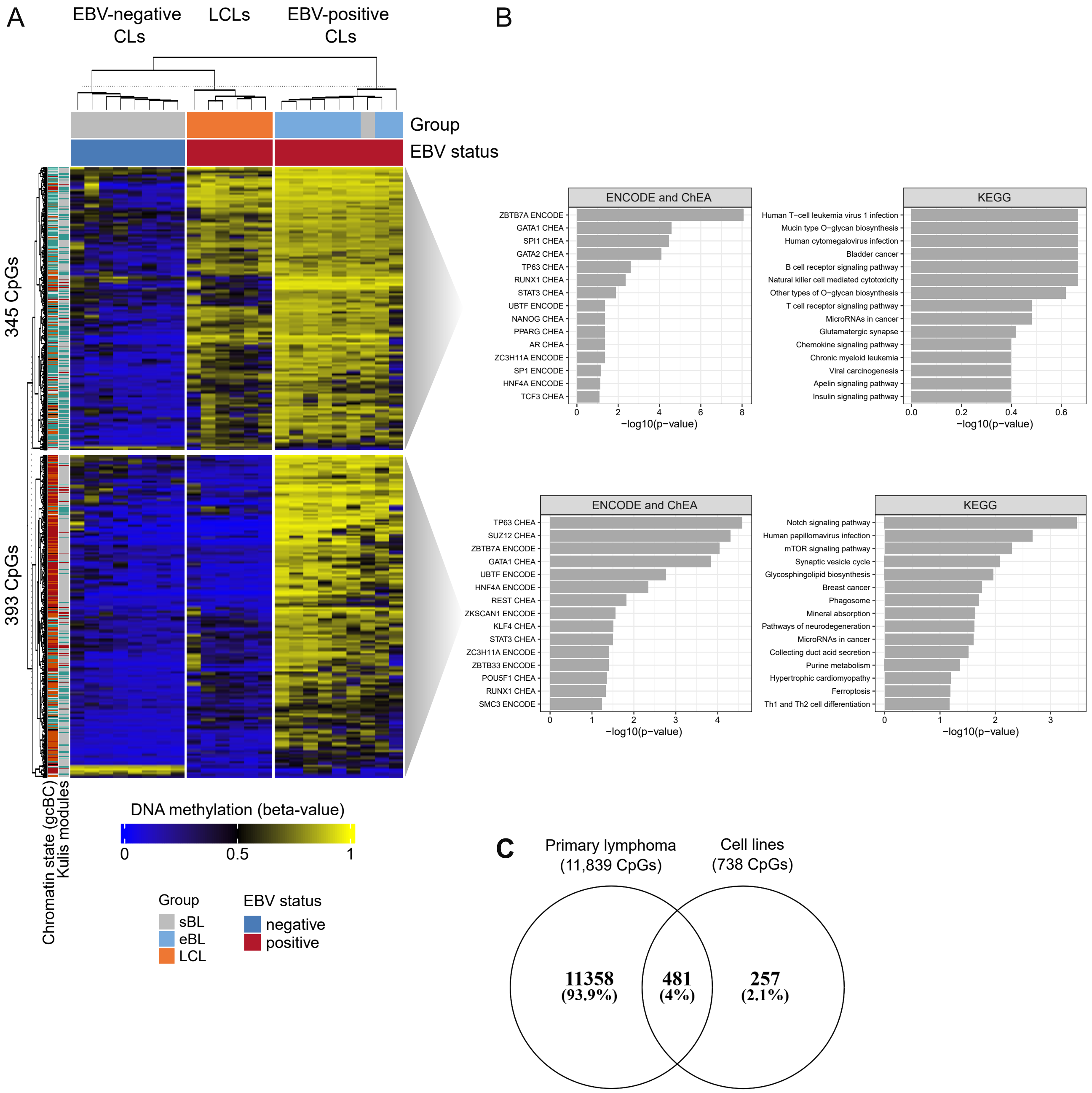

Supplementary Figure S9: Differential DNA methylation analysis of EBV-positive and EBV-negative Burkitt lymphoma cell lines. A: Heatmap displaying DNA methylation levels of 738 differentially methylated CpGs (adjusted p-value < 0.01, |Δβ| > 0.2). Lymphoblastoid cell lines were excluded from analysis and solely displayed. Rows represent CpGs and column represent samples. Both samples and CpGs are clustered with k-means clustering, resulting in two distinct CpG modules. **B:** Enrichment analysis results for the two CpG modules mentioned in (A). The analysis includes transcription factor binding sites (ENCODE and ChEA databases) and biological pathways (KEGG database). **C:** Venn diagramm showing the overlap of the significant differentially methylated CpGs between EBV-positive and EBV-negative cases in primary lymphoma and cell lines.

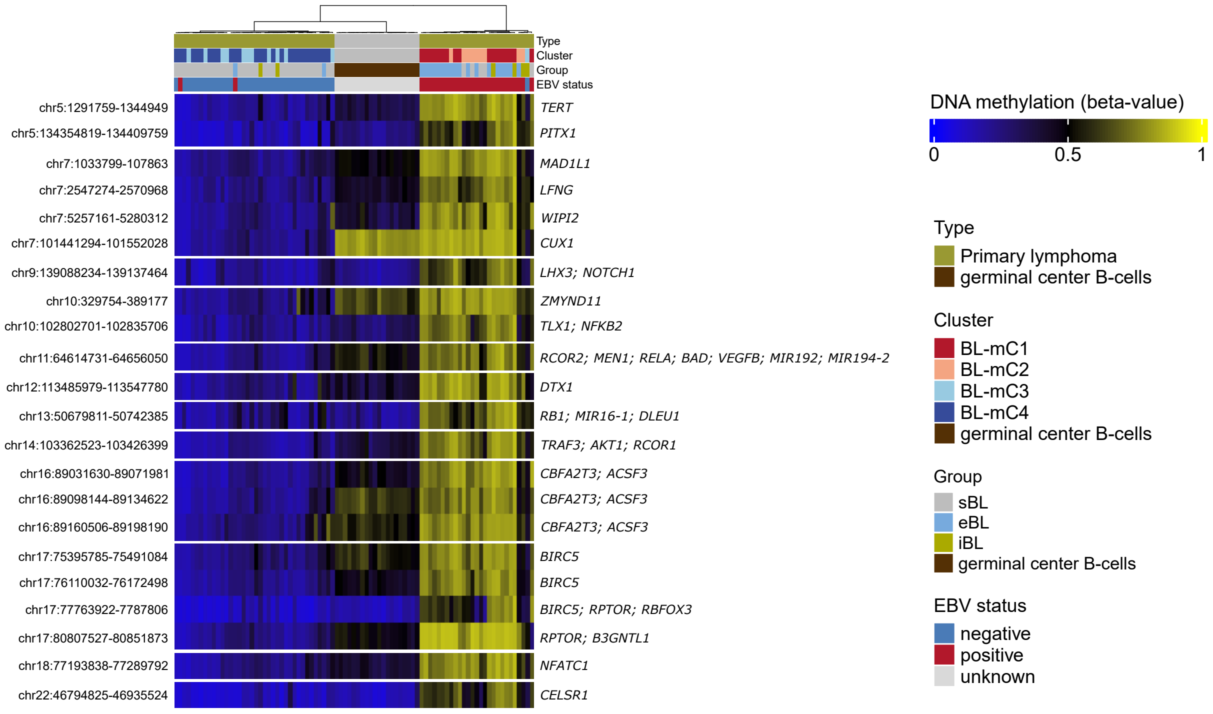

Supplementary Figure S10: DNA methylation patterns of superenhancers in Burkitt lymphoma subtypes. The heatmap displays DNA methylation levels of 22 superenhancers affected by differential DNA methylation between EBV-positive and EBV-negative Burkitt lymphoma cases. Only superenhancers affected by at least 10 differentially methylated CpGs are shown (adjusted p-value < 0.01, $|\Delta\beta| > 0.3$). Median DNA methylation levels were calculated across the CpGs for each superenhancer. Superenhancers are grouped according to chromosomes. The left panel displays genomic coordinates for each superenhancer. The right panel lists selected genes potentially impacted by the enhancer, located within a ±2 megabase genomic range.

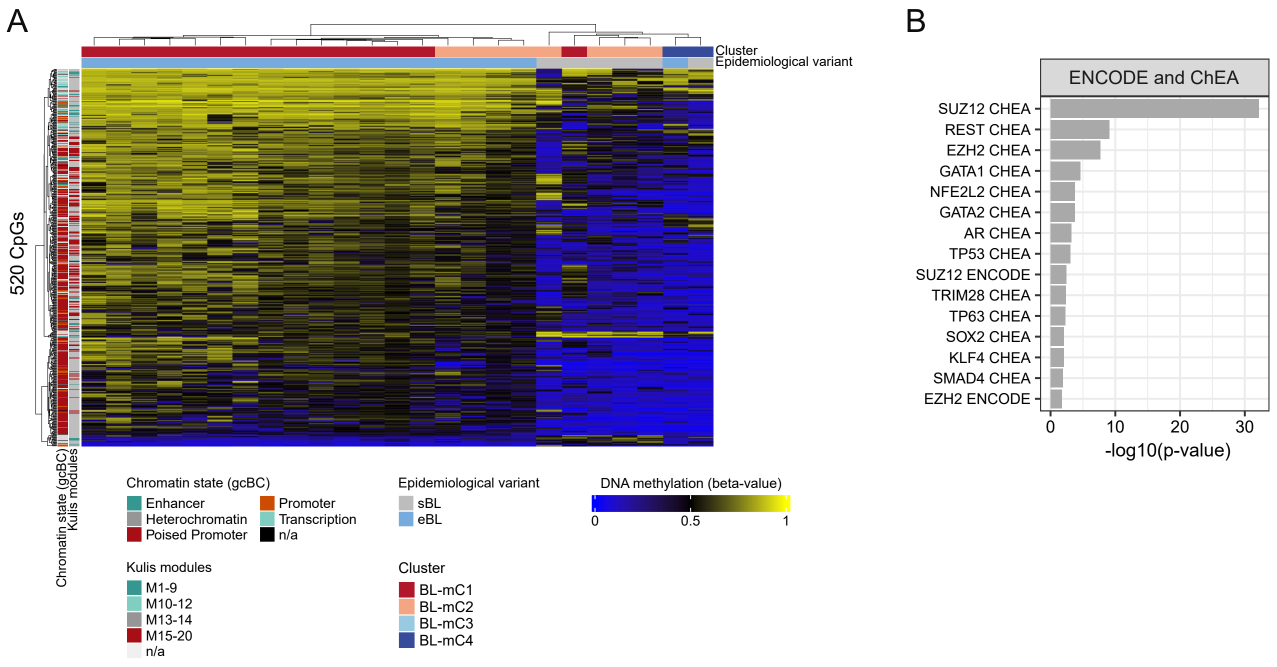

Supplementary Figure S11: Differential DNA methylation analysis of EBV-positive endemic and sporadic Burkitt lymphoma. A: Heatmap displaying DNA methylation levels of 520 differentially methylated CpGs (adjusted p-value < 0.01, $|\Delta\beta| > 0.2$) between EBV-positive eBL (n=17) and sBL (n=5). The two cases in which EBV infection was likely a bystander than a driver event (Cluster BL-mC4), were excluded from the differential DNA methylation analysis. Rows represent CpGs and column represent samples. **B:** Transcription factor enrichment analysis (ENCODE and ChEA) on the genes associated with the 520 CpGs.

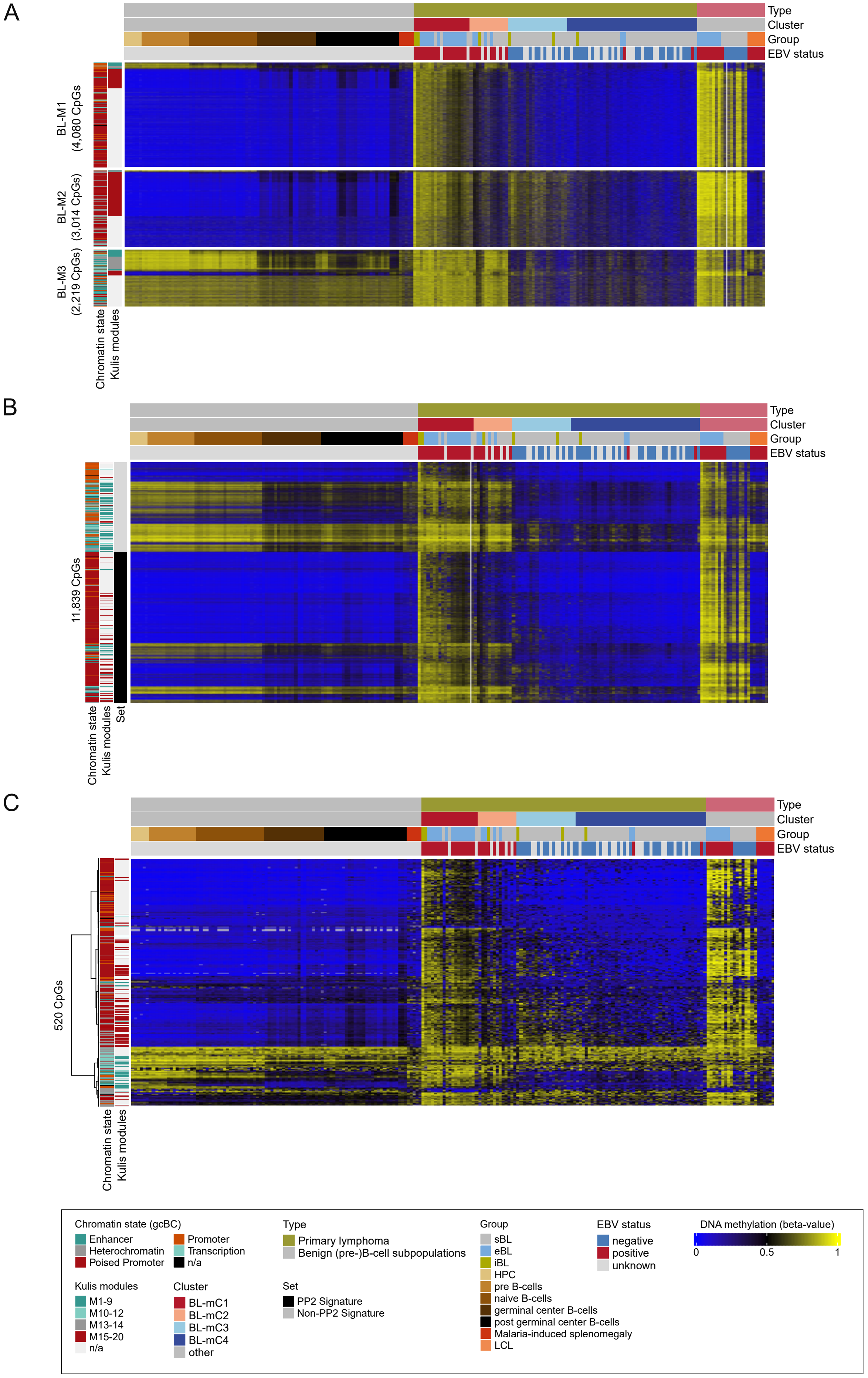

Supplementary Figure S12: Comparison of DNA methylation levels with malaria-induced splenomegaly samples. Heatmaps showing DNA methylation levels of the 9,313 CpGs (A) adapted from Figure 1C, 11,839 CpGs (B) adapted from Figure 2B, and 520 CpGs (C) from Supplementary Figure S11 expanded to incorporate non-malignant (pre-)B-cell subpopulations (n = 93), cell lines (n = 23) as well as malaria-induced splenomegaly (n = 5). Rows represent individual CpGs, and columns represent samples. PP2 Signature, Non-PP2 Signature. PP2: Poised promoter and polycomb repressive complex 2; GC: germinal center; n/a: not applicable.
